# Satellite RNAs promote pancreatic oncogenic processes via the dysfunction of YBX1

**DOI:** 10.1038/ncomms13006

**Published:** 2016-09-26

**Authors:** Takahiro Kishikawa, Motoyuki Otsuka, Takeshi Yoshikawa, Motoko Ohno, Hideaki Ijichi, Kazuhiko Koike

**Affiliations:** 1 Department of Gastroenterology, Graduate School of Medicine, The University of Tokyo, Tokyo 113-8655, Japan; 2 Japan Science and Technology Agency, PRESTO, Kawaguchi, Saitama 332-0012, Japan

## Abstract

Highly repetitive tandem arrays at the centromeric and pericentromeric regions in chromosomes, previously considered silent, are actively transcribed, particularly in cancer. This aberrant expression occurs even in K-ras-mutated pancreatic intraepithelial neoplasia (PanIN) tissues, which are precancerous lesions. To examine the biological roles of the satellite RNAs in carcinogenesis, we construct mouse PanIN-derived cells expressing major satellite (MajSAT) RNA and show increased malignant properties. We find an increase in frequency of chromosomal instability and point mutations in both genomic and mitochondrial DNA. We identify Y-box binding protein 1 (YBX1) as a protein that binds to MajSAT RNA. MajSAT RNA inhibits the nuclear translocation of YBX1 under stress conditions, thus reducing its DNA-damage repair function. The forced expression of YBX1 significantly decreases the aberrant phenotypes. These findings indicate that during the early stage of cancer development, satellite transcripts may act as ‘intrinsic mutagens' by inducing YBX1 dysfunction, which may be crucial in oncogenic processes.

## Abstract (web summary)

Satellite RNAs are aberrantly transcribed during pancreatic cancer progression. Here, the authors show that major satellite RNA binds to the multifunctional protein YBX1 in the cytoplasm and induces mutations in nuclear and mitochondrial DNA by preventing YBX1 stress-induced intracellular translocation.

## Graphical abstract

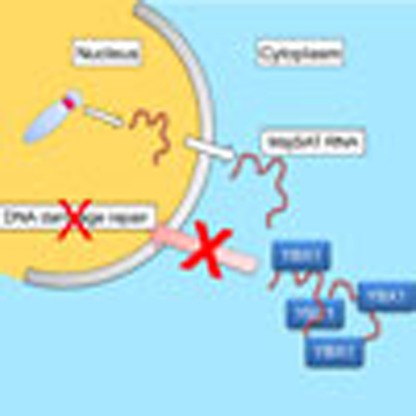

Pancreatic cancer, one of the most intractable diseases, develops in incremental steps with the sequential activation of oncogenes and the dysfunction of tumour suppressor genes^1^,^2^. However, the frequently mutated genes are relatively limited, such as KRAS, TP53, CDKN2A, SMAD4 (refs 3, 4, 5, 6, 7). In particular, constitutively active mutations of the K-ras gene are observed in almost all pancreatic cancers (>95%) and are found in 36–87% of pancreatic intraepithelial neoplasia (PanIN) tissues, which are considered to be the precancerous lesions of the pancreatic cancer^6^,^7^,^8^. These observations might indicate that mutations in K-ras occur during the earlier stage of pancreatic PanIN-carcinoma sequence, and the accumulation of mutations in other genes during the later stage causes the cellular transformation. These hypotheses are supported by the fact that genetically engineered mice with pancreas-specific K-ras mutation form regional PanIN-like lesions via acinar-to-ductal metaplasia, whereas the additional deletion of tumour suppressor genes, such as TP53, SMAD4 or TGFR2, causes the development of invasive cancer^1^,^9^.

Satellite DNAs, which consist of highly repetitive non-coding sequences in huge monomeric arrays, are largely located in the centromeric and pericentromeric regions of the chromosomes. These chromosomal structures are conserved in almost all eukaryotes, although each monomeric sequence is different between species^10^. In the mouse genome, the centromeric region consists of 120-base monomeric arrays, called minor satellites, and the pericentromeric region is composed of 234-base monomeric arrays, called ‘major satellites (MajSATs)'. Previously, satellite regions were believed to be silent because of their constitutive heterochromatin structures. However, recent studies have provided evidences that these regions are actively transcribed^11^. Some reports have shown that the appropriate transcription of these satellite regions is essential for accurate cell division^12^,^13^,^14^, heterochromatin establishment in mouse embryonic development^15^,^16^ and cell differentiation^17^. In contrast to these physiological roles, the aberrant transcription of satellite sequences can be observed in epithelial tumours, especially in pancreatic cancers including PanIN lesions^18^. While the overexpression of satellite RNAs may cause mitotic errors, such as centrosome amplification and incorrect separation, or genomic DNA damage, such as double-strand breaks^15^,^19^,^20^, the pathological roles of these aberrantly expressed satellite RNAs, especially in precancerous tissues, are not yet fully determined.

Y-box binding protein 1 (YBX1) is a multifunctional protein, mainly known as a transcriptional and translational regulator that is involved in DNA repair, centrosome maturation and mRNA splicing^21^,^22^. This protein is typically localized to the cytoplasm and acts as an RNA-binding protein^23^. However, when cells are exposed to stress conditions, such as oxidative stress and ultraviolet irradiation, YBX1 often translocate into the nucleus^24^,^25^. Nuclear YBX1 has been considered to participate in DNA-damage repair activity via diverse but currently undefined mechanisms^22^.

In this study, we confirmed that MajSAT RNA is expressed in precancerous PanIN lesions *in vivo*. This finding led us to hypothesize that the aberrant expression of MajSAT RNA contributes molecularly to malignant transformation. We show that aberrantly expressed MajSAT RNA in PanIN-derived cells increases the chromosomal instability and mutations in genomic and mitochondrial DNAs (mtDNA), by inducing YBX1 dysfunction. The increased instability and mutations due to satellite RNAs may molecularly explain the process of the transformation of precancerous cells to invasive cancers.

## Results

### Expression of MajSAT RNA in mouse pancreatic cells

**Figure 1:**
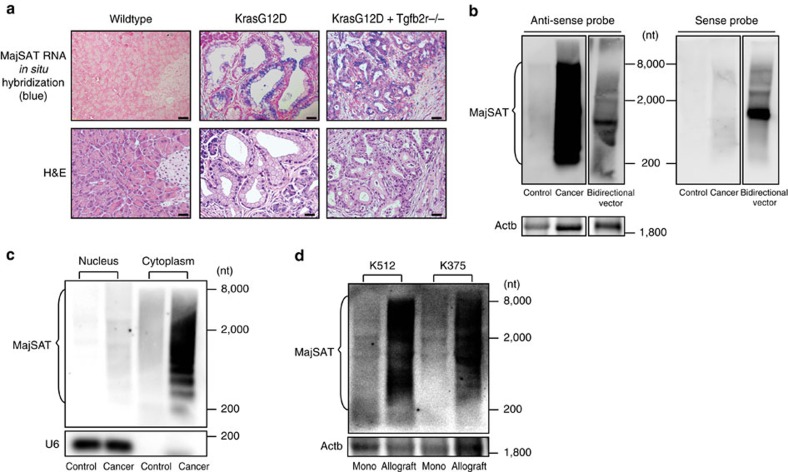
MajSAT RNA expression in pancreatic tumours. (**a**) Expression of MajSAT RNA was determined in pancreatic control, PanIN and carcinoma tissues by RNA *in situ* hybridization. The tissues were derived from wildtype, KrasG12D and KrasG12D+Tgfbr2^−/−^ mice, respectively. Representative images of two independent experiments are shown. Upper panels: blue, MajSAT RNA; red, nucleus. Lower panels: hematoxilin and eosin staining. Bar, 50 μm. (**b**) Forward strand of MajSAT RNA is dominantly expressed in pancreatic cancer tissue. RNAs extracted from pancreatic tissues from wildtype mice (Control) and pancreatic cancerous tissues from KrasG12D+Tgfbr2^−/−^ mice (Cancer) were subjected to northern blotting. Antisense probe and sense probe were used to detect the forward and reverse strands of MajSAT RNA, respectively. As a control, RNAs after transiently transfecting 293TN cells with vector bidirectionally expressing MajSAT (pBI-CMV1-MajSAT-Fw-Rv) were also applied. β-actin (Actb) was re-probed using the same membrane to confirm almost equal loading. nt, nucleotides. Representative results from three independent experiments are shown. (**c**) MajSAT RNA is localized mainly to the cytoplasm of pancreatic cancer tissues. RNAs extracted from the nuclear and cytoplasmic fractions of pancreatic control and cancerous tissues were subjected to northern blotting. U6 expression was confirmed as a nucleus marker. Representative results from three independent experiments are shown. (**d**) MajSAT RNA expression in tumor-derived cell lines is lost in monolayer culture *in vitro*. Two types of cell lines established from pancreatic cancers from KrasG12D, and KrasG12D+Tgfbr2^−/−^ mice, K512 and K375 cells, were long-term cultured in plastic dishes or subcutaneously transplanted into nude mice for 3 weeks. RNAs extracted from the indicated sources were subjected to northern blotting. Mono: monolayer cultured cells *in vitro*, Allograft: transplanted cells. Representative results from two independent experiments are shown.

First, we examined the MajSAT RNA expression status in two types of genetically induced mouse pancreatic tumour models. Mice with constitutively active K-ras specifically in the pancreatic cells (KrasG12D mice) spontaneously develop PanIN lesions resembling human PanIN tissues^9^. Mice with constitutively active K-ras and Tgfb2r deletion specifically in the pancreatic cells (KrasG12D+Tgfbr2^−/−^ mice) develop aggressive pancreatic carcinoma at 6–7 weeks of age^9^. Consistently with the previous report^18^, MajSAT RNA was expressed in cancer cells as well as in PanIN cells, while no MajSAT RNA expression was observed in wildtype tissues (Fig. 1a).

To further characterize the MajSAT RNA expression in pancreatic cancerous cells, we performed northern blotting using a probe containing a single 234 bp array of the MajSAT repeat consensus sequences. Because the transcript lengths of MajSAT RNA were highly heterogeneous, they were detected as smear or ladder bands covering from ∼200 to 8,000 nucleotide, which was consistent with other reports^18^,^26^ (Fig. 1b). In addition, northern blotting using strand-specific probes revealed that MajSAT RNA was transcribed exclusively from the forward strand of the genomic DNA, similarly to human satellite III (satIII) RNA^27^, which is expressed under limited circumstances in a strand-specific manner from pericentromeric satellite sequences^28^,^29^. The expressed MajSAT RNA in cancerous cells was localized mainly to the cytoplasm when testing the fractioned RNAs (Fig. 1c). Interestingly, consistently with the previous reports^18^,^20^, MajSAT RNA expression could not be detected in K512 and K375 cells, established cell lines from PanIN and pancreatic cancer cells in the mice described above, respectively, when they were cultured as a monolayer in a dish. However, MajSAT RNA was re-expressed when the cells were transplanted subcutaneously onto the backs of nude mice as allografts (Fig. 1d).

### MajSAT causes chromosomal and genomic instability

**Figure 2:**
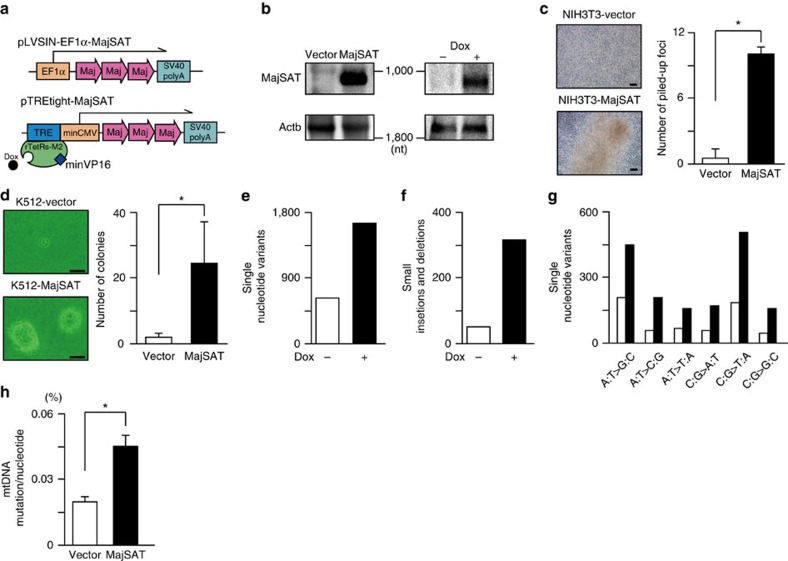
MajSAT RNA causes chromosomal and genomic instability. (**a**) Two constructs expressing MajSAT RNA used are shown. EF1α promoter-driven constitutively MajSAT RNA-expressing plasmid (upper; EF1α-MajSAT) and tetracycline-regulated MajSAT RNA-expressing plasmid (lower; TREtight-MajSAT), for which the MajSAT RNA expression can be shut off by removing tetracycline. Both constructs express approximately three tandem repeats of the 234 nucleotide basic unit of MajSAT RNA. Dox, doxycycline; Maj, MajSAT basic unit. (**b**) Confirmation of MajSAT RNA expression in K512 cells stably transfected with EF1α-MajSAT (left) or TREtight-MajSAT (right) by northern blotting. Dox+, doxycycline at 1 μg ml^−1^ for 48 h. nt, nucleotides. Representative results from three independent experiments are shown. (**c**) Focus formation assay of NIH3T3 stably MajSAT RNA-expressing (NIH3T3-MajSAT) cells. The number of piled-up foci that escaped from contact growth inhibition was counted after 14 days culture. Representative cell images are shown at left. Bar, 50 μm. Data on the number of foci represent the mean±s.e. of three independent experiments (right). *P<0.05 (Welch's *t* test). (**d**) Soft-agar colony formation assay using K512 stably expressing MajSAT RNA (K512-MajSAT) cells. Representative cell images are shown (left). Bar, 50 μm. The number of colonies grown to over 50 μm in size was counted and summarized (right). Data represent the mean±s.e. of four independent experiments. *P<0.05 (Welch's *t* test). (**e**,**f**) MajSAT RNA expression increases genomic instability. Whole-exon sequences in K512-TREtight-MajSAT cells after culturing with or without doxycycline for 4 weeks were examined by high-throughput sequencing. The total numbers of acquired somatic mutations (**e**) and small insertions/deletions (**f**) in each group are shown. (**g**) The spectrum of acquired somatic mutations. The frequencies of the indicated base change patterns are shown. White and black bars indicate cells cultured without and with doxycycline, respectively. (**h**) Frequencies of mutations in the mtDNA. The cloned libraries of the D-loop region of mtDNA, generated from DNA extracts of K512-vector (vector) and K512-MajSAT (MajSAT) cells, were sequenced. Mutation frequencies out of total sequenced nucleotides are summarized. Data represent the mean±s.e. of two independent experiments. *P<0.05 (Student's *t* test).

As described above, MajSAT RNA was expressed in a strand-specific manner in precancerous cells *in vivo*, but the expression was not observed in tumour-derived cell lines *in vitro*. Utilizing these results, to characterize the biological effects of the aberrant MajSAT RNA expression in precancerous cells *in vitro*, we established constitutively MajSAT RNA-expressing cells. We constructed two types of expression constructs: pLVSIN-EF1α-MajSAT, which constitutively expresses forward-strand MajSAT RNA driven by the EF1α promoter, and pTREtight-MajSAT, which expresses forward-strand MajSAT RNA only in the presence of doxycycline (Fig. 2a). Because it is practically difficult to express all lengths of the satellite repeats simultaneously by the currently available methods, approximately three tandem repeats of the basic unit sequences of MajSAT RNA were chosen for expression in this study (Fig. 2a) to examine the effects of the basic sequence and of the junctional sequences between the units. We confirmed the stable expression and the tightly regulated expression by doxycycline, respectively, in the construct transduced cells (Fig. 2b).

Using the NIH3T3 and the PanIN-derived K512 cells stably expressing MajSAT RNA, we first performed a focus formation assay and a soft-agar colony formation assay. In both cases, cells with MajSAT RNA expression showed higher rates of the acquisition of malignant phenotypes, escape from contact-inhibition and acquirement of anchorage-independent growth (Fig. 2c,d), although the cell proliferation rate was not changed by the expression of MajSAT RNA (Supplementary Fig. 1a,b). Similar to previous observations using primary human mammary epithelial cells (HMECs)^19^, the frequencies of mitotic errors, such as spindle multipolarity, micronuclei and anaphase bridging, during cell division were significantly increased by MajSAT RNA expression (Supplementary Fig. 1c–f). While these chromosomal instabilities may lead to losses, gains or translocations of chromosomes, which are observed in pancreatic cancer cells, small mutations at the nucleotide level, such as base substitutions and the insertion or deletion of nucleotides, are crucial in the pathogenesis of PanIN-carcinoma sequence^3^,^4^,^5^,^7^. Thus, we next performed comprehensive exome sequencing to examine whether MajSAT RNA expression enhances the spontaneous mutation rate. To equalize the starting mutation levels, K512 cells expressing MajSAT regulated by doxycycline were used for this test. After culturing the cells with or without doxycycline for 4 weeks, exome sequencing was performed to compare the rates of single nucleotide variants (snv) and of small insertions and deletions (indel) in cells with and without MajSAT RNA expression (Supplementary Data 1 and 2; Fig. 2e–g). The results showed that MajSAT RNA expression increased the snv and indel rates (Fig. 2e,f), while the spectra of base alternations were comparable in both cell types, suggesting that more DNA damage (or DNA-damage repair impairment) occurred to the bases universally in cells expressing MajSAT RNA (Fig. 2g).

Moreover, because mtDNA is generally more sensitive and mutagenic under genotoxic stress than is genomic DNA^30^, we also examined the changes in the mutation rates in mtDNA by MajSAT RNA expression. We sequenced the non-coding D-loop region of mtDNA, which was reported to have a high mutation tendency^31^. The frequencies of point mutations and small nucleotide insertions/deletions were significantly higher in K512-EF1α-MajSAT cells (Fig. 2h; Supplementary Figs 2 and 3), suggesting that MajSAT RNA significantly disturbs the intracellular homoeostasis by enhancing the genomic instability in both nuclear and mtDNA, which may contribute to the pro-carcinogenetic steps in the long-term.

### MajSAT RNA is specifically bound to YBX1 protein

**Figure 3:**
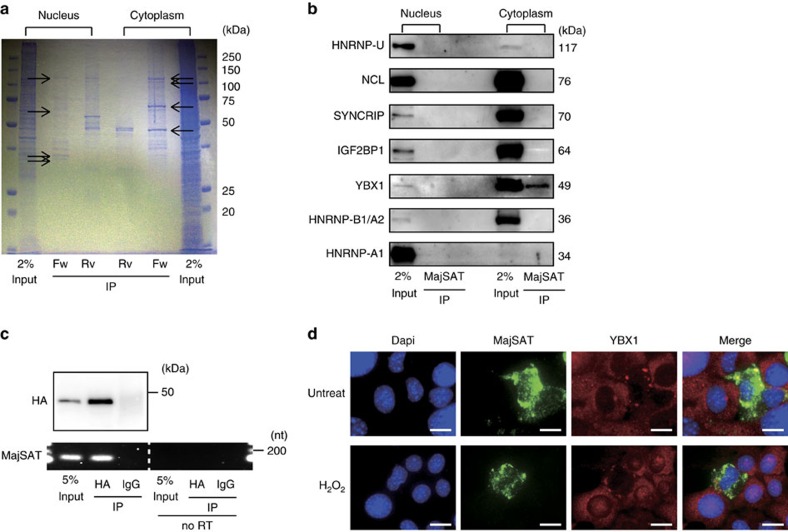
MajSAT RNA is specifically bound to YBX1 protein. (**a**) Nuclear and cytoplasmic extracts from K512 cells were incubated with BrU-labelled *in vitro* transcribed MajSAT forward (Fw) RNA and, as a control, reverse (Rv) RNA *in vitro*, followed by immunoprecipitation with anti-BrU antibody. Bound proteins were separated and stained by Coomassie Brilliant Blue. Arrowed bands, which specifically bound with forward-strand MajSAT RNA, were cut out and identified by LC–MS/MS. Input: 2% nuclear or cytoplasmic extract of K512 cells, Rv: MajSAT reverse-strand probe, Fw: MajSAT forward-strand probe. (**b**) Confirmation of the possible binding proteins for MajSAT RNA. Immunoprecipitated nuclear and cytoplasmic proteins with BrU-labelled MajSAT Fw RNA were immunoblotted with antibodies against indicated proteins, which were identified as candidate proteins by mass spectrometry. Representative results from two independent experiments are shown. (**c**) MajSAT Fw RNA was transiently transfected into stably HA-tagged YBX1 overexpressing K512 cells (K512-HAYBX1). RNA immunoprecipitation with anti-HA tag antibody conjugated with agarose beads was performed. The binding of MajSAT RNA to HA-tagged YBX1 protein was determined by RT-PCR. Upper panels: western blotting using immunoprecipitates after anti-HA or control IgG immunoprecipitation. Lower panels: semi-quantitative RT-PCR images of MajSAT RNA. Five per cent of the cell lysates were used as controls (input). As a negative control, samples that were not reverse transcribed are shown on the right side of the panel (no RT). Representative results from two independent experiments are shown. (**d**) Partial co-localization of MajSAT RNA and YBX1 protein. K512 cells were transiently transfected with pLVSIN-EF1α-MajSAT plasmid and treated with or without 300 μM of H_2_O_2_ for 6 h. MajSAT RNA was detected by *in situ* hybridization, and YBX1 was subsequently immunostained. MajSAT RNA-overexpressing cells and untransfected cells are intentionally shown in the same field of view for comparison. Representative images from five independent experiments are shown. Bar, 10 μm.

To determine the possible molecular mechanisms of the genomic instability induced by MajSAT RNA expression, we searched for proteins interacting with MajSAT RNA, as non-coding RNAs frequently function by interacting with specific proteins^32^,^33^. Nuclear and cytoplasmic lysates from K512 cells were mixed with BrU-labelled forward- (as samples) and reverse-strand (as negative controls) MajSAT RNA, followed by immunoprecipitation. After electrophoresis, specific bands observed only in the samples derived from the forward RNA were excised (Fig. 3a) and analyzed by liquid chromatography-tandem mass spectrometry (LC–MS/MS) (Supplementary Table 1). Candidate proteins were subsequently validated by western blotting using specific antibodies. Through these processes, only YBX1 was confirmed to be a MajSAT RNA-binding protein, observed in the cytoplasmic fraction (Fig. 3b). Intracellular binding was also confirmed by detecting MajSAT RNA using anti-HA antibodies in HA-tagged YBX1 immunoprecipitates from HA-YBX1-expressing K512 cells transduced with MajSAT RNA (Fig. 3c).

Although YBX1 localizes to the cytoplasm under normal conditions, nuclear translocation was reported under various stress conditions such as oxidative stress, ultraviolet irradiation and the treatment with DNA-damage inducing agents^22^. Several reports showed that translocated YBX1 enhances DNA-damage repair machinery by interacting with DNA-damage repair genes^24^,^34^. Because MajSAT RNA is expressed in the cytoplasm, as determined by the northern blot results shown above, and binds to YBX1 in the cytoplasm, we hypothesized that the translocation of YBX1 into the nucleus was inhibited by its trapping in the cytoplasm by MajSAT RNA. First, fluorescence *in situ* hybridization (FISH) analysis using K512 cells transiently transfected with a MajSAT RNA-expressing plasmid (pLVSIN-EF1α-MajSAT) confirmed the primarily cytoplasmic distribution of MajSAT RNA. Probe specificity was confirmed by the disappearance of the targeted RNAs following RNase treatment (Supplementary Fig. 4). Next, to determine the intracellular localization of MajSAT RNA and YBX1 protein, we performed double staining of MajSAT RNA and YBX1 protein by *in situ* hybridization and immunofluorescence staining using K512 cells transiently transfected with MajSAT RNA-expressing plasmid (pLVSIN-EF1α-MajSAT), which enabled us to compare MajSAT RNA-expressing and non-expressing cells in the same field of view. MajSAT RNA was localized mainly to the cytoplasm, which was consistent with the northern blotting results (Fig. 1c). Although YBX1 was diffusely distributed in the cytoplasm in the cells not transfected with MajSAT RNA, YBX1 was aggregated into particles in the cytoplasm and co-localized with MajSAT RNA in MajSAT RNA-expressing cells even without any other stimulation (Fig. 3d, upper panels). Furthermore, the nuclear translocation of YBX1 under oxidative stress induced by H_2_O_2_ treatment was inhibited in MajSAT RNA-expressing cells (Fig. 3d, lower panels). The percentages of nuclear translocation of YBX1 were 6.7±3.6% in MajSAT RNA-expressing cell and 74.5±8.2% in non-expressing cells, respectively (mean±s.e. from the results of twenty fields observation). These phenomena were similarly observed after DNA-damage induction by ultraviolet irradiation (Supplementary Fig. 5a,b). To confirm the specificity of YBX1 retention by MajSAT RNA, K512 cells expressing reverse-strand MajSAT RNA (MajSAT Rv) were also established as a negative control (Supplementary Fig. 6a). Interestingly, unlike sense MajSAT RNA, antisense MajSAT RNA localized to the nucleus (Supplementary Fig. 6b), consistent with a previous report^15^, and did not impair YBX1 translocation to the nucleus (Supplementary Fig. 6c). Nuclear translocation of proteins known as YBX1-interacting proteins, RBBP6, CTCF1, ANKRD2 and p53 (ref. 22), was also evaluated following H_2_O_2_ treatment. Among the proteins analyzed, RBBP6 translocation to the nucleus after stress induction was impaired (Supplementary Fig. 7a–d). This result suggests that the impaired nuclear transport of YBX1 by MajSAT RNA could affect the nuclear transport of YBX1-interacting proteins, ultimately affecting their intracellular localization.

### MajSAT RNA impairs DNA-damage repair activity

**Figure 4:**
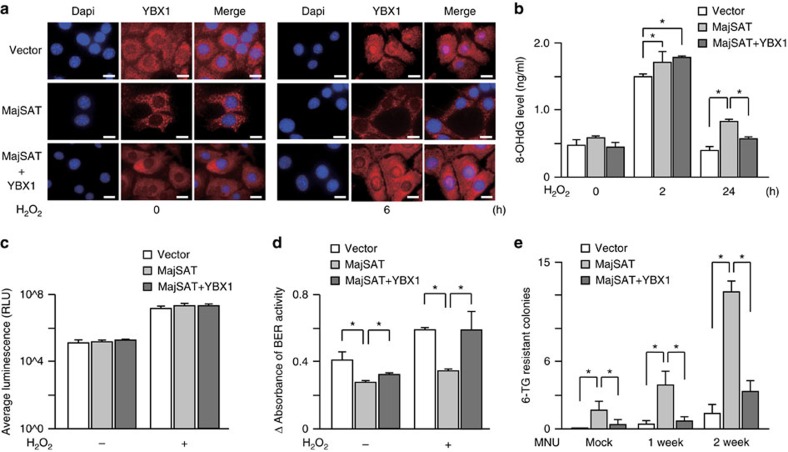
MajSAT RNA reduces DNA repair function via YBX1. (**a**) MajSAT RNA reduces YBX1 movement into the nucleus after oxidative stress, which is recovered by the forced expression of YBX1. K512 cells stably expressing MajSAT RNA or MajSAT RNA with the forced expression of YBX1 were used. Cells were treated with or without 400 μM H_2_O_2_ for 6 h. The nuclear entry of YBX1 was determined by immunofluorescence staining. Representative images from three independent experiments are shown. Bar, 10 μm. (**b**) The recovery of oxidative DNA damage was delayed in MajSAT RNA-expressing cells (MajSAT), which was countered by the forced expression of YBX1 (MajSAT+YBX1). The levels of 8-OHdG were determined by competitive ELISA after the treatment of cells with 400 μM of H_2_O_2_ for the indicated periods. Data represent the mean±s.e. of three independent experiments. *P<0.05 (Student's *t* test). (**c**) Cellular ROS levels were not significantly changed by MajSAT RNA or YBX1 expression. Average luminescences, reflecting cellular ROS levels, were measured using K512 cells before and after 400 μM H_2_O_2_ stimulation for 2 h. (**d**), BER activity was lower in MajSAT RNA-expressing cells, which was rescued by YBX1 forced expression. The 8-OHdG excision activity was determined by the colorimetric DNAzyme assay. To determine the response to oxidative stress, cells were incubated in 400 μM H_2_O_2_ for 4 h. Data represent the mean±s.e. of three independent experiments. *P<0.05, (Student's *t* test). (**e**) Spontaneous missense mutations in the HPRT gene occurred more frequently in MajSAT RNA-expressing K512 cells. Cells were treated with 2 μM MNU for 1 or 2 weeks to promote the tendency for mutation and subsequently cultured in 6-TG-containing media. The number of surviving colonies, in which mutations in the HPRT gene presumably occurred, was counted. Data represent the mean±s.e. of three independent experiments. *P<0.05 (Welch's *t* test).

To examine whether the increased mutation rates in MajSAT RNA-expressing cells were due to the increased DNA damages by the inhibition of the intra-nucleus YBX1 function, we established MajSAT RNA-expressing K512 cells with overexpression of GFP-tagged YBX1 (K512-MajSAT-YBX1GFP; Supplementary Fig. 8a,b) to overcome the binding of YBX1 to MajSAT RNA in the cytoplasm. The expression levels of YBX1 protein were approximately doubled in these cells (Supplementary Fig. 8a), and the nuclear translocation of YBX1 was rescued by H_2_O_2_ treatment was rescued (Fig. 4a). Using these cells, we measured cellular levels of 8-hydroxy-2′-deoxyguanosine (8-OHdG), the most commonly used indicator of oxidative DNA damage, before and after H_2_O_2_ treatment. The 8-OHdG levels at 2 and 24 h after H_2_O_2_ treatment were significantly increased in MajSAT RNA-expressing cells, whereas the effect was negated at 24 h by YBX1 protein overexpression (Fig. 4b).

Because of this time course and because the cellular reactive oxygen species (ROS) levels were not significantly changed in these cells (Fig. 4c), we hypothesized that the increased 8-OHdG levels in MajSAT RNA were caused by the impairment and retardation of DNA-damage repair. Because 8-OHdG is repaired mainly by the base excision repair (BER) pathway^35^,^36^, we determined the BER activity using the colorimetric DNAzyme-based assay^37^,^38^. BER activity in the MajSAT-expressing K512 cells was significantly reduced even in the steady condition. Furthermore, BER activities were enhanced in K512-vector control cells after H_2_O_2_ treatment, while such enhancement was hardly observed in MajSAT RNA-expressing cells (Fig. 4d).

To further examine the rescue effects of YBX1 overexpression in MajSAT-expressing cells, we performed the hypoxanthine phosphoribosyl transferase (HPRT) mutation assay. In this assay, once cells acquire HPRT gene missense mutations, they survive and form colonies under selection by 6-thioguanine (6-TG), which is converted into cell-toxic 6-mercaptopurine by functional HPRT. As expected, 6-TG resistant colonies increased in direct proportion to the mutagenic MNU treatment period (Fig. 4e). However, the number of surviving colonies expressing MajSAT RNA was significantly higher and the phenomenon was clearly rescued by YBX1 expression (Fig. 4e).

Finally, we observed the reduced copy numbers of mtDNA (mt-Co1 and mt-Cytb) in MajSAT RNA-expressing cells, reflecting the increased mtDNA mutations^39^, which were also rescued by YBX1 protein overexpression (Supplementary Fig. 8c). All of these results suggested that MajSAT RNA expression impairs intrinsic YBX1 function and leads to an increase in the number of genomic and mtDNA mutations.

To exclude the possibility that the effects of MajSAT RNA harbouring only three repeats of the basic unit were specific to that particular construct, we generated K512 cells expressing MajSAT RNA with six repeats (Supplementary Fig. 9a) and confirmed elevated 8-OHdG levels, increased numbers of 6-TG resistant colonies and decreased copy numbers of mtDNA as a result of genomic instability (Supplementary Fig. 9b–d). Although this does not reproduce the endogenous heterogeneous conditions, these results suggest that the basic units of MajSAT RNA are likely responsible for the phenotypes caused by MajSAT RNA shown here.

### MajSAT RNA-non-interacting mutant recovers YBX1 function

To further confirm the biological significance of the YBX1-MajSAT RNA interaction, we constructed a series of flag-tagged YBX1 deletion constructs to determine the domain of YBX1 responsible for its interaction with MajSAT RNA (Supplementary Fig. 10a,b). We found that the C terminus (amino acids 218–323) of YBX1 is crucial for its interaction with MajSAT RNA, because the flag-tagged C-terminal YBX1 deletion mutant (hereafter referred to as FmYBX1) no longer interacted with MajSAT RNA, according to RNA immunoprecipitation (Supplementary Fig. 10b). To replace the wildtype YBX1 with FmYBX1 in K512 cells, siRNAs targeting the 5′ untranslated region of YBX1 were transfected into K512 cells stably expressing FmYBX1, resulting in efficient knock down of endogenous wildtype YBX1 but not FmYBX1 (Supplementary Fig. 10c). The nuclear translocation of FmYBX1 following H_2_O_2_ treatment was retained in these cells (Supplementary Fig. 10d). Furthermore, reduced BER activity, increased 8-OHdG levels, increased 6-TG resistant colonies and decreased mtDNA levels were recovered in FmYBX1- and MajSAT RNA-expressing cells (Supplementary Fig. 10e–h). The results observed with the MajSAT RNA-non-interacting mutant of YBX1 suggest that the MajSAT RNA–YBX1 interaction impairs YBX1 function, resulting in an increased number of genomic and mtDNA mutations.

## Discussion

Herein, we show that MajSAT RNA is aberrantly expressed in mouse PanIN (mPanIN) cells carrying a constitutively active K-ras gene, and it increases the number of genomic and mtDNA mutations by binding with YBX1, in addition to increasing the mitotic instability. All of these effects may promote the cellular transformation of precancerous cells.

The regulatory mechanisms of satellite transcription from the centromeric and pericentromeric genomic regions, which are normally in a heterochromatin state, are not yet fully clarified. Although it is suggested that epigenetic alternations in cancer cells such as global DNA hypomethylation may induce aberrant transcription from heterochromatin regions^26^,^40^,^41^, this hypothesis still remains controversial^42^,^43^. Nonetheless, aberrantly high expression levels of satellite RNAs have been reported in various types of epithelial cancers, including pancreatic cancer and colon cancer, both of which have higher rates of K-ras gene mutations^18^,^20^. Importantly, the aberrant expression of satellite RNAs was also observed in PanIN tissues^18^. Consistent with previous reports, we also detected aberrantly high expression levels of MajSAT RNA in mouse PanIN tissues with genetically induced K-ras mutations. Although a few reports have demonstrated possible regulator of satellite transcription, such as Snail1/LOXL2 (ref. 44), p53 (ref. 40) or Pax3 and Pax9 (ref. 45), K-ras mutations are apparently among the potent inducers of aberrant MajSAT RNA transcription while the downstream events are not fully clarified.

Aberrantly highly expressed satellite RNAs in precancerous cells may have oncogenic potential in the sequential carcinogenesis model. Consistent with a previous report in which the loss of BRCA1 in mammalian cancer cells causes derepression of satellite RNAs by H2A monoubiquitination and induces centrosome amplification^19^, MajSAT RNA overexpression did induce chromosomal instability, such as spindle multipolarity, micronuclei and anaphase bridging. In addition, we determined that MajSAT RNA expression might induce the acquisition of somatic mutations in the genome, possibly resulting in the occurrence of mutations in the defined driver genes during the sequential carcinogenesis process. These results, together with the very recent findings that satellite RNA transcripts stimulate the production of proinflammatory cytokines^46^ and that human satellite II (HSATII) transcripts lead to the progressive elongation of pericentromeric regions by intrinsic reverse transcriptases^20^, may indicate that aberrant satellite RNA expression plays more crucial roles in long-term carcinogenesis than previously considered.

Another important finding in this study is that the aberrant expression of satellite RNA increases mutations in mtDNA. As recently reported in the *Drosophila* model, epithelial cells that are defective in mitochondrial function in conjunction with oncogenic Ras expression potently induced tumour progression in the surrounding tissues^47^. The high rates of mitochondrial malfunction in cancers are caused by somatic mutations in the mtDNA^48^,^49^, which are frequently identified in pancreatic cancers^50^, and the accumulation of mtDNA mutations is already apparent in precancerous lesions^30^. From these results, the aberrant expression of MajSAT RNA in PanIN cells might drive not only cell-autonomous but also non-autonomous carcinogenesis in the neighbouring cells via these mechanisms.

We identified YBX1 as a binding protein of MajSAT RNA. Although some previous reports demonstrated that satellite transcripts are normally localized to the nucleus^45^,^51^, we found that aberrantly expressed MajSAT RNA is located mainly in the cytoplasm *in vivo* and *in vitro* and impairs the translocation of YBX1 into the nucleus. While intracellular localization of some YBX1-interacting proteins, such as RBBP6, is also affected by MajSAT RNA expression, YBX1 is one of the key factors for genomic DNA-damage repair^22^. Various mechanisms for DNA repair by YBX1 have been suggested, such as endonuclease activity^52^,^53^, the transcriptional enhancement of repair genes^54^,^55^, the enhancer of DNA glycosylation to excise damaged nucleotide bases^24^,^34^ and cooperation with p53 in the nucleus^56^. In addition, YBX1 is also a key molecule for mtDNA-damage repair, in which fewer numbers of molecules participate compared with genomic DNA repair^57^,^58^. Therefore, it is speculated that the effects of YBX1 dysfunction are more severe in mtDNA repair than genomic DNA repair. This difference may be reflected by our results indicating that the copy number of mitochondrial DNA was significantly lower in MajSAT-expressing cells, most likely due to the rapid removal of deteriorated mitochondria^59^, which also plays an active role in the aetiology of cancer according to Warburg's hypothesis^47^.

In this study, we found that MajSAT RNA is distributed primarily in the cytoplasm. There are inconsistencies among subcellular distribution analyses; however, several reports have identified satellite RNA in the nucleus^13^,^20^, and others have identified it in both the nucleus and cytoplasm^15^,^28^. During the developmental stages, nuclear and cytoplasmic distribution of MajSAT RNA has been reported^15^, and it is noteworthy that only the sense sequences were found in both the cytoplasm and nucleus, while antisense transcripts were found only in the nucleus. Although the reasons for these discrepancies are unknown, it will be important to exclude the possibility of genomic DNA cross-hybridization when MajSAT RNA is observed in the nucleus, since MajSAT RNA is encoded by multiple regions throughout the genome. In addition, differences in methods used for *in situ* hybridization, including fixation, permeabilization and proteinase treatment, may affect the ability to determine the distribution of MajSAT RNA.

We also found that the sense sequences of MajSAT RNA are expressed in pancreatic cancerous tissues. While accumulation of satellite RNAs in both orientations have been observed in other conditions^45^,^51^, previous reports have also shown that strand-specific transcription of major satellite RNA occurs during the 2-cell stage of the mouse embryo^15^, and an increase in forward-strand satIII transcription was observed following genotoxic stress in HeLa cells^28^,^29^. Because the expression of satellite RNAs varies, the cellular conditions used in these studies may explain some of the discrepancies. Further experiments addressing the orientation and intracellular distribution of MajSAT RNAs, as well as the expression levels and heterogeneity of satellite RNAs, will be necessary to interpret these results fully.

In this study, we expressed MajSAT RNAs with three or six repeats of the consensus sequence and assessed their biological function. The endogenous MajSAT RNAs, however, are more heterogeneous with additional repeats. While there is currently no adequate method to express all heterogeneous MajSAT RNAs *in vitro*, our results may suggest that the basic components of the repeat sequences alone have pathological effects even at lower expression levels.

In summary, our findings reveal that aberrantly expressed satellite transcripts may act as ‘intrinsic mutagens' and have oncogenic potential. Although satellite sequences are not conserved among eukaryotes, and it still remains unclear which sequences in the human genome are the actual counterparts of mouse MajSAT; the sequences HSATII and satIII, which are also located in the pericentromeric region and are aberrantly transcribed in human PanIN tissues^18^, may be such candidates^43^. Further elucidating their deregulated expression mechanisms and the downstream events may provide unexpected clues toward the prevention of carcinogenesis.

## Methods

### Mouse and cell lines

*Tgfbr2*^*flox/flox*^ (ref. 60), *Ptf1a*^*cre/+*^(ref. 61) and *LSL-Kras*^*G12D/+*^ (ref. 62) mouse lines were intercrossed to generate *Ptf1a*^*cre/+*^; *LSL-Kras*^*G12D/+*^; *Tgfbr2*^*flox/flox*^ (KrasG12D+Tgfbr2^−/−^) and *Ptf1a*^*cre/+*^; *LSL-Kras*^*G12D/+*^ (KrasG12D) mice in a >95% C57BL/6 background^9^. All of the experimental protocols were approved by the internal ethics committee for animal experimentation and conducted in accordance with the Guidelines for the Care and Use of Laboratory Animals of the Graduate School of Medicine, University of Tokyo (Tokyo, Japan).

Mouse primary pancreatic cells derived from mPanIN, which is considered the adenomatous premalignant state of pancreatic tissue, and pancreatic cancer tissues were established from 9-week-old male KrasG12D mouse (K512 cells) and 8-week-old male KrasG12D+Tgfbr2^−/−^ mouse (K375 cells), respectively, as previously described^9^,^63^,^64^. Briefly, freshly isolated tumour specimens were cut with sterile razor, digested with dispase II (Invitrogen, Carlsbad, CA, USA)/collagenase (Invitrogen) (4 mg ml^−1^ each) for 1 h at 37 °C, and then resuspended in Roswell Park Memorial Institute (RPMI) media supplemented 20% fetal bovine serum and seeded on vitrogen/fibronectin coated plates. After the cells reached pure population, cells were cultured in collagen-coated dishes in RPMI with 10% bovine serum. NIH3T3 and 293TN cell lines were purchased from the American Type Culture Collection (ATCC, Manassas, VA, USA) and System Biosciences (SBI, Mountain View, CA, USA), respectively. These cells were cultured in Dulbecco's Modified Eagle's Medium (DMEM) media supplemented with 10% fetal bovine serum. All cells were incubated at 37 °C, 20% O_2_ and 5% CO_2_.

### Subcutaneous allograft model

For allogenic transplantation, 1 × 10^5^ of K512 and K375 cells in 150 μl of RPMI media was mixed with 100 μl of Matrigel basement membrane matrix, LDEV-Free (Corning, Corning, NY, USA) and immediately injected subcutaneously into the backs of BALB/cByJJcl mice (CREA Japan, Tokyo, Japan). The resulting tumours were excised after 4 weeks.

### Plasmids

MajSAT sequences were obtained from a 788 bp array at chromosome 2: 98506702:98507489 (Build: UCSC mm9), which contains almost three tandem repeats of mouse major satellites, as described previously^18^. The polymerase chain reaction (PCR) product was cloned into pCR2.1-TOPO vector (Invitrogen) by the TA cloning method using One Shot DH5α TOP10 competent cells (Invitrogen). The primers used were as follows: Fw: 5′-CGTTTCCAACGAATGTGTTT-3′ and Rv: 5′-TGGAAACAGATGATTTCGTC-3′. The ligated plasmids were named pCR2.1-MajSAT-Fw for the forward-strand insertion and pCR2.1-MajSAT-Rv for the reverse-strand insertion.

The pLVSIN-EF1α-MajSAT vector was constructed as follows. MajSAT sequences in the pCR2.1-MajSAT-Fw/Rv vector were digested with EcoRI restriction enzyme, subcloned into the pCDH-CMV-MCS-EF1-Puro vector (SBI) (named as pCDH-MajSAT-Fw/Rv vector) and subsequently subcloned into the pLVSIN-EF1α Pur vector (TaKaRa, Shiga, Japan) by digestion at the XbaI and NotI sites. To append the polyA signal downstream of the MajSAT sequences, SV40 polyA signal sequences from pCDH-CMV-MCS-EF1-Puro vector at the positions of 4354–5656 were amplified by PCR and cloned into the pLVSIN-EF1α pur vector at the NotI site using the InFusion HD cloning kit (Clontech, Mountain View, CA, USA) according to the manufacturer's protocol (named as pLVSIN-MajSAT-Fw/Rv vector). The primers used were as follows: Fw: 5′-ATCGGATCCGCGGCCGCCAGTGTGGAAAATCTCTAGCAG-3′, Rv: 5′-AGATCCTTCGCGGCCGCAAAATTAGTCAGCCATGGGGC-3′.

The pTREtight-MajSAT vector was generated by subcloning MajSAT sequences from the pCR2.1-MajSAT-Fw vector into the pTRETight vector (Clontech) at the EcoRI site. The pBI-CMV1-MajSAT-Fw-Rv vector was constructed using the pBI-CMV1 vector (Clontech). MajSAT Fw sequences digested with BamHI and EcoRV were subcloned into the multi-cloning site (McsI) site, and MajSAT Rv sequences digested with BglII were subcloned into the McsII site of the pBI-CMV1 vector.

To double the number of MajSAT unit sequences (MajSAT-double), the MajSAT-Fw sequence was subcloned into the pLVSIN-MajSAT-Fw vector at the XbaI site using the InFusion HD cloning kit. The primers used were as follows: Fw: 5′-GGATTTAAATTCTAGTCTAGAGCTAGCGAATTCG-3′ and Rv: 5′-ATTCGCTAGCTCTAGCGAATTCGCCCTTTGGAAA-3′.

To construct probe templates for northern blotting and RNA *in situ* hybridization, 234 bp of consensus mouse major satellite sequences referred from RepBase (http://www.girinst.org/repbase/index.html) were cloned into the pCR2.1-TOPO vector by TA cloning (named pCR2.1-MajSAT230Fw and pCR2.1-MAjSAT230Rv). The primers used were as follows: Fw: 5′-GGACCTGGAATATGGCGAGAA-3′ and Rv: 5′-TTCAGTGTGCATTTCTCATTT-3′. For the β-actin control probe, the mouse Actb open reading flame was cloned by TA cloning using the cDNA of K512 cells. The primers used were as follows: Fw: 5′-GACGAGGCCCAGAGCAAGAG-3′, and Rv: 5′-TGCGCTCAGGAGGAGCAATG-3′.

For the overexpression of YBX1 protein, the pCMV6kan-mYBX1-cDNA vector (MC204526) was purchased from OriGene Technologies (Rockville, MD, USA). Using this plasmid as a template, PCR-amplified HA-tagged YBX1 ORF was subcloned into the pCDH-CMV-MCS-EF1-Neo vector (pCDH-HA-YBX1). To express YBX1 protein conjugated with GFP protein (pLVSIN-YBX1-GFP), the pCMV6-mYBX1-cDNA-GFP vector (MG204659, OriGene) was purchased and subcloned into the pLVSIN-EF1α neo vector (TaKaRa) at the BamHI site using the InFusion HD cloning kit. The primers used were as follows: Fw: 5′-GAGCGGCCGCGGATCCGGTACCGAGGAGATCTGC-3′, and Rv: 5′-CGGTAGAATTGGATCCGTTTAAACTCTTTCTTCACCG-3′.

### Transfection and lentivirus transduction

Transient transfection of the plasmids was performed using FuGENE HD Transfection Reagent (Promega, Madison, WI, USA). Briefly, 24 h after 1 × 10^5^ cells were seeded in a 6-well dish, 1 μg of template vector and 3 μl FuGENE Reagent diluted in serum-free media were added. To generate stably expressing polyclonal cells, Lentivirus Packaging System (SBI) was used according to the manufacturer's protocol. Briefly, 1 μg of MajSAT- or YBX1-overexpressing vector and 5 μg of pPACKH1 packaging plasmid mix were transfected into 293TN cells using Effectene Transfection Reagent (Qiagen, Hilden, Germany). After 24 h, the collected culture media was mixed with one-fifth the volume of PEG-it Reagent (SBI) overnight at 4 °C to concentrate the viruses. The centrifuged pellet was resuspended in 1 × PBS, and aliquots were stored at −80 °C. Viruses were added to the target cells with Polybrene Reagent (Santa Cruz Biotechnology, Dallas, TX, USA) and after 48 h, antibiotic selection (3 μM puromycin or 150 μM neomycin) was begun.

To generate Tet-induced MajSAT-expressing cells, K512 cells were first transfected with the pTet-On-advance vector (Clontech) following 150 μM neomycin selection. Tet-ON-advanced cell clones were subsequently transfected with the pTREtight-MajSAT vector along with the Linear Hygromycin Marker (Clontech). MajSAT RNA expression after induction by doxycycline (1 μg ml^−1^) was confirmed in the clones selected with 150 μM hygromycin.

For transfection of siRNAs targeting YBX1, the pre-designed TriFECTa Dicer-Substrate RNAi Kit for mouse YBX1 (Integrated DNA Technologies, Coralville, IA, USA) was purchased, and 5 × 10^4^ cells were transfected with 10 nmol of each siRNA oligo in 12-well plates using Lipofectamine RNAiMAX Transfection reagent (Thermo Fisher, Waltham, MA, USA) according to the manufacturer's protocol. The siRNA sequences were as follows: siRNA#1: 5′-UUGGUCAUCCAACAAGAAGAAAUGA-3′, and siRNA#2: 5′-AAUCAAGGAGAUGAGACCCAAGGTC-3′.

### Probe synthesis

For northern blotting and RNA *in situ* hybridization of mouse pancreatic tissues, MajSAT230Fw, MajSAT230Rv and β-actin RNA probes were synthesized from the pCR2.1-MajSAT230Fw, pCR2.1-MAjSAT230Rv and pCR2.1-mActb-cDNA vectors by *in vitro* transcription using MEGAscript T7 Kit (Ambion, Austin, TX, USA), followed by biotin labelling using the Psoralen-Biotin Kit (Ambion). Biotin-labelled U6 snRNA probe was synthesized as an RNA oligo probe (Eurofins Genomics, Tokyo, Japan).

### RNA *in situ* hybridization of mouse pancreatic tissues

Mouse pancreatic tissues were resected from 26-week-old male wildtype mouse, 28-week-old male KrasG12D mouse and 8-week-old male KrasG12D+Tgfbr2^−/−^ mouse. Paraffin-embedded mouse pancreatic tissues were deparaffinized by three treatments with Histo-Clear (IWAKI, Tokyo, Japan) and subsequently incubated in a series of 100, 95 and 70% ethanol. Slides were washed for 3 min in 1 × PBS and incubated with 5 μg ml^−1^ Proteinase K solution for 10 min at 37 °C, followed by post-fixation with 4% paraformaldehyde (PFA). After washing twice with 1 × PBS, antigen retrieval was performed by delivering acetic acid anhydride by drops into 0.1 M Tris–HCl buffer (pH 8). After washing with 4 × saline sodium citrate (SSC), the slides were dehydrated with 70, 95 and 100% ethanol. Next, 50 ng ml^−1^ of biotin-labelled MST-Rv probe, 500 μg ml^−1^ of yeast tRNA and 100 μg ml^−1^ salmon DNA in the hybridization buffer (5 × SSC, 50% formamide) were applied to the slide glass and incubated overnight at 50 °C. After stringent washing with 5 × SSC for 5 min, 1 × SSC for 5 min and 0.1 × SSC for 5 min at 50 °C, the slides were blocked and stained using the DIG Wash & Block Kit (Roche Diagnostics, Basel, Switzerland). Briefly, slides were blocked for 30 min with 1 × DIG block buffer and incubated with 0.2 μg ml^−1^ Streptavidin-Alkaline Phosphatase (Ambion) diluted in 1 × block buffer for 30 min. After washing three times with 1 × wash buffer, the targets were then visualized using NBT/BCIP solution (Sigma-Aldrich, St Louis, MO, USA) with levamisole (Dako, Glostrup, Denmark) for 10 min and counterstained with Nuclear Fast Red (Sigma-Aldrich) for 1 min.

### RNA extraction

Mouse pancreatic tissue was immediately frozen by liquid nitrogen after resection and stored at −80 °C. Frozen tissues were crushed without thawing by the SK mill (Tokken, Chiba, Japan) and immediately immersed in ice-cold Isogen reagent (Nippon gene, Tokyo, Japan).

Nuclear and cytoplasmic RNA were isolated separately using the Cytoplasmic & Nuclear RNA purification kit (Norgen Biotek, Thorold, ON, USA) according to the manufacturer's protocol.

### Northern blotting

Northern blotting was performed as described previously^65^ with slight modification. Briefly, 5 μg of tissue RNAs were separated in 1% formaldehyde denatured agarose gel and hydrostatically transferred to Hybond N+ membrane (GE Healthcare, Chalfont St Giles, UK). Membranes were ultraviolet-crosslinked and prehybridized in the hybridization buffer. Hybridization was performed overnight at 42 °C in ULTRAhyb Buffer (Ambion) containing 10 ng ml^−1^ of biotin-labelled RNA probe, which had been denatured at 90 °C for 10 min. Membranes were stringently washed at 55 °C in 2 × SSC containing 0.1% SDS and in 0.1 × SSC containing 0.1% SDS, twice each, and the bound probe was visualized using a BrightStar BioDetect Kit (Ambion), according to the manufacturer's protocol.

### Focus formation and soft-agar colony formation assay

To examine their ability to escape from contact-inhibition, NIH3T3 cells on a 10 cm dish were maintained in confluency for 2 weeks, changing the media at every two days. Piled-up foci were counted by crystal violet staining. To evaluate the acquisition of anchorage-independent growth, 1.5 × 10^4^ of K512 cells were added to 1.5 ml of plating agar, consisting of 1 × RPMI and 10% FBS containing 0.35% agarose (Sigma-Aldrich), and poured onto a base RPMI agar containing of 0.5% agarose in a 6-well dish. Colonies grown to over 50 μm in size were counted after 3 weeks of incubation.

### Exome sequencing

K512-TREtight-MajSAT cells were cultured in RPMI media with and without 1 μg ml^−1^ of doxycycline and passaged every 3 days for 4 weeks. DNA was extracted using a QIAamp DNA mini kit (Qiagen). Three micrograms of qualified genomic DNA was fragmented by an ultrasonicator Covaris S-series (Covaris, Woburn, MA, USA). The fragments were purified, end blunted, ‘A' tailed, and adaptor ligated using the Agilent SureSelectXT Human All Exon v4 Kit (Agilent Technologies, Santa Clara, CA, USA) in accordance with the SureSelectXT Target Enrichment for Illumina Multiplexed Sequencing Protocol. Five cycles of PCR were performed after size selection in the gel. Then, 750 ng aliquots of these purified libraries were hybridized to the SureSelect oligo probe capture library (Agilent) for 24 h. After hybridization, washing and elution with magnetic beads, the eluted fraction was PCR-amplified for 12 cycles with index primer, purified and quantified using quantitative PCR and an Agilent 2100 Bioanalyzer (Agilent) to obtain sufficient DNA template for the downstream applications. Paired end, 100-bp read-length sequencing was performed by the HiSeq 2000 according to the manufacturer's instructions (Illumina, San Diego, CA, USA). After removing reads containing sequencing adaptors and low-quality reads with more than five ambiguous bases, high-quality reads were aligned to the UCSC mouse reference genome (mm9) using BWA (v0.5.9) with default parameters. Picard (v1.7.0) (http://picard.sourceforge.net/) was used to mark duplicates. Somatic point mutations and somatic indels were detected by VarScan2.3.2 and SAMtools (mpileup, v0.1.18)^66^ with default parameters^67^. ‘Somatic' mutation was defined as mutations acquired in MajSAT RNA-expressing cells. ‘LOH' was defined as mutations acquired in cells not expressing MajSAT RNA. Detected snv and indel mutations were annotated using the snpEff tool (v3.0f)^68^.

### Oxidative and ultraviolet stress

For oxidative stress induction, 400 μM H_2_O_2_ (Wako Pure Chemical Industries Ltd., Osaka, Japan) was added to the culture media and washed out twice with the normal media to remove H_2_O_2_. Ultraviolet irradiation was performed by a Stratalinker ultraviolet Crosslinker (Stratagene, La Jolla, CA, USA) preceded by two washes with 1 × PBS to remove the culture media.

### Cell growth

For the cell growth assay, the Cell Counting Kit-8 (Dojindo Molecular Technologies, Kumamoto, Japan) was used according to the manufacturer's protocol. Briefly, 1 × 10^4^ cells were seeded in 96-well plates. Then, 10 μl of the CCK-8 solution was added, and the plates were incubated for 90 min at 37 °C, followed by measurement of the absorbance at 450 nm using a microplate reader (Bio-Rad Laboratories, Hercules, CA, USA).

### mtDNA mutation

To examine the mutation rate in the mitochondrial DNA, a 431 bp fragment of the D-loop region was cloned using the InFusion HD cloning kit (Clontech) as follows. Total DNA templates including mtDNA were isolated from K512-vector and K512-EF1α-MajSAT cells using the QIAamp DNA Mini Kit (Qiagen), and PCR was performed with LA-Taq polymerase (TaKaRa) mixed with Pfu turbo DNA Polymerase (Stratagene). The primers used were as follows: Fw: 5′-CTGGACTAGTGGATCCTCTTTTTATTTTGGCCTACTTT-3′, Rv: 5′-TACCGAGCTCGGATCCCATCTAAGCATTTTCAGTGC-3′ (ref. 31). The pcDNA 3.1(−) vector was digested at the BamHI site and dephosphorylated with Shrimp Alkaline Phosphatase (TaKaRa) at 37 °C for 15 min. Amplified fragments and linearized vector were incubated with 5 × InFusion HD Enzyme premix at 50 °C for 15 min and subsequently transformed into ECOS Competent *E. coli* DH5α. After 16 h, at least twenty colonies from each sample were picked up, and cloned vectors were collected using the QIAprep 96 Turbo Miniprep Kit (Qiagen). The inserted D-loop sequences of the cloned libraries were determined using a T7 sequence primer (5′-TAA TAC GAC TCA CTA TAG GG-3′). The mutations were determined by comparison with the referee sequences (GenBank: NC_005089.1).

### RNA immunoprecipitation

To screen MajSAT RNA-binding protein *in vitro*, nuclear and cytoplasmic cell lysates were separated from K512 cells using the RiboTrap Kit (Medical & Biological Laboratories, MBL, Nagoya, Japan). Briefly, 3 × 10^7^ cells on three 10 cm dishes were harvested and lysed in 1,200 μl of CE buffer and 60 μl of detergent solution. After centrifugation at 3,000*g* for 3 min at 4 °C to precipitate the nuclei, the supernatant was collected, and 36 μl of high-salt solution was added, followed by centrifugation at 12,000*g* for 3 min at 4 °C. The supernatant was collected as the cytoplasmic extract. The pellet was washed three times with 1 ml of CE Wash buffer and resuspended in 500 μl of NE buffer followed by a quick sonication. Homogenized samples were added to 700 μl of dilution buffer and centrifuged at 16,000*g* for 10 min at 4 °C. The supernatant was used as the nuclear extract. To generate BrU-labelled MajSAT-Fw and MajSAT-Rv probes for RNA immunoprecipitation, RNA probes were prepared by *in vitro* transcription using the MEGAscript T7 kit (Ambion) with a 1:3 molar ratio of 5-bromo-UTP to standard UTP. Each 50 pmol of RNA probe was bound to anti-BrU antibody-conjugated Protein G agarose beads. The nuclear and cytoplasmic extracts were precleared with Protein G agarose and subsequently mixed with BrU-labelled MajSAT-Fw or MajSAT-Rv probes for 2 h at 4 °C. RNA-binding protein/BrU-labelled RNA complexes were washed with wash buffer and eluted using spin columns to eliminate the agarose beads. The eluates and 2% inputs were separated on 5–20% gradient polyacrylamide gel by SDS–PAGE followed by Coomassie Brilliant Blue (CBB) staining. Representative bands specifically detected on the MajSAT-Fw lanes were excised from the gel and analyzed by LC–MS/MS. The resulting tryptic peptides were separated and analyzed using reversed phase capillary HPLC directly coupled to a Finnigan LCQ ion trap mass spectrometer (LC–MS/MS) with a slight modification. The individual spectra from MS/MS were processed using the TurboSEQUEST software (Thermo Quest, San Jose, CA, USA). The generated peak list files were used to query either the MSDB database or NCBI using the MASCOT programme (http://www.matrixscience.com).

To examine the endogenous binding of YBX1 protein to MajSAT RNA, the RIP assay microRNA kit (MBL) was used according to the manufacturer's protocol. Briefly, 6 × 10^6^ K512-HAYBX1 cells were seeded on a 10 cm dish, and 8 μg of BrU-labelled MajSAT-Fw RNA was transfected into the cells using TransMessenger reagent (Qiagen). After 8 h incubation, cells were harvested and lysed, followed by the addition of precleared Protein G agarose beads for 1 h. The supernatants were mixed with agarose beads conjugated to anti-HA tag-antibody or control rabbit IgG for 4 h at 4 °C. The pellets were washed four times, and bound RNA was isolated by ethanol precipitation. To detect bound MajSAT RNA, 1 μg of precipitated RNA and 5% input were reverse transcribed to cDNA using SuperScript III Reverse Transcriptase (Invitrogen), and semi-quantitative RT-PCR was performed using Mighty Amp DNA polymerase (TaKaRa). The primers used were Fw: 5′-ACCCAAGCTGGCTAGCGTT-3′ and Rv: 5′-TTCTTTCCAAAGTAGGTACACACAC-3′.

### Western blot analysis and antibodies

Western blotting was performed as previously described^69^. Briefly, lysate samples were separated on 5–20% gradient polyacrylamide gel by SDS–PAGE following electrical transfer to PVDF membranes (GE Healthcare). After blocking with 5% dry milk, membranes were probed with the appropriate primary antibodies diluted in Immunoshot Reagent 1 (Cosmo bio, Tokyo, Japan) overnight at 4 °C. HRP-conjugated corresponding secondary antibodies (GE Healthcare) were subsequently used. Trueblot anti-rabbit IgG HRP (Rockland, Limerick, PA, USA, 1:1,000) was used as the secondary antibody to avoid interfering immunoprecipitated immunoglobulin heavy and light chains. Bound antibodies were detected using Immunostar LD reagents (Wako). The following antibodies were used: Hnrnp-U (#ab180952, 1:1,000) and Nucleolin (#ab22758, 1:1,000) from Abcam (Cambridge, UK); HA tag (#561, 1:10,000) and Syncrip (#RN046PW, 1:1,000) from MBL; Hnrnp-A2/B1 (#R4653, 1:1,000) and β-actin (#A1978, 1:2,000) from Sigma-Aldrich; and YBX1 (D299, 1:1,000), Igf2BP1 (D33A2, 1:1,000) and HnrnpA1 (D21H11, 1:1,000) from Cell Signaling Technology (CST, Danvers, MA, USA).

### Immunofluorescence imaging

Cells were seeded in 4-well glass chambers (IWAKI) with manual collagen coating. Cells were fixed with 4% PFA in 1 × PBS for 15 min at RT and permeabilized with 0.1% Triton-X in 1 × PBS for 20 min at room temperature. Primary antibodies diluted by 1:100 with Can Get Signal Immunostaining Solution A (Toyobo, Osaka, Japan) were applied to the samples and incubated overnight at 4 °C. Cells were incubated with the second antibodies conjugated with Alexa 555 or 488 (Molecular Probe, Eugene, OR, USA, 1:500) for 1 h at room temperature and mounted using fluorescence mounting medium with DAPI (Dako). Images were captured under an Olympus DP72 microscope digital camera system using the Olympus DP2-TWAIN software. The antibodies used were as follows: YBX1 (D299, CST, 1:100), α-tubulin (#3873, CST, 1:100), γ-tubulin (#T6557, Sigma-Aldrich, 1:100), RBBP6 (#NBP1–49535, Novus Biologicals, Littleton, CO, USA, 1:100), CTCF (#3418, CST, 1:400), ANKRD2 (#sc-138111, Santa Cruz Biotechnology, 1:100) and p53 (sc-6243, Santa Cruz Biotechnology, 1:100). Full-length blot images are available in Supplementary Fig. 11.

### RNA *in situ* hybridization of cultured cells

The intracellular localization of MajSAT RNA was visualized using the RNA Scope Fluorescent Multiplex Reagent Kit (Advanced Cell Diagnostics (ACD), Hayward, CA, USA). The probes for sense and antisense MajSAT RNA were designed by the ACD website (http://www.acdbio.com/) using mouse major satellite consensus sequences. First, 1 × 10^5^ cells that had been transiently transfected by with pLVSIN-MajSAT-Fw or pLVSIN-MajSATRv vector were plated in collagen-pre-coated 4-well glass slide chambers (IWAKI). Cells were fixed with 4% PFA at 4 °C for 20 min, followed by gradual dehydration with 70, 90 and 100% ethanol for 5 min. As a negative control, 10 μg ml^−1^ ribonuclease solution (Wako) was added to the slides and incubated for 30 min at 37 °C, followed by two washes with 2 × SSC. After a short Proteinase K treatment with 0.2 × Pretreat3 Reagent for 15 min at RT, the slides were hybridized with MajSAT Rv probe for 2 h at 40 °C in the HybEZ Hybridization System (ACD). Subsequently, the slides were reacted with Amplifier #1 to #4 for 15–30 min at 40 °C and washed twice with 1 × wash buffer. For the double staining of MajSAT RNA and YBX1 protein, washed slides were subsequently incubated with anti-YBX1 antibody (CST) diluted by 1:100 for 1 h at RT, followed by the protocol described in the immunofluorescence imaging section.

### 8-OHdG measurement

To evaluate intracellular oxidative stress levels, 8-OHdG was measured by ELISA using the High Sensitive ELISA kit for 8-OHdG (Japan Institute for the Control of Aging (JaICA), Shizuoka, Japan). Briefly, 6 × 10^6^ cells were cultured on a 10 cm dish and treated with 400 μM H_2_O_2_ for the indicated periods. DNA was extracted using the DNA extractor TIS kit (Wako) and fragmented and dephosphorylated using the 8-OHdG assay preparation reagent set (JaICA) according to the manufacturer's protocol. Then, 100 μg of heat-denatured DNA was attached to each well of the plates, and 8-OHdG in the samples or standards was probed with anti-8-OHdG antibody, followed by incubation with HRP-conjugated secondary antibody. The 8-OHdG levels in the samples were determined by comparison with a standard curve. The absorbance was measured at 450 nm by a microplate reader.

### Cellular ROS measurement

Cellular ROS levels were measured using the ROS-Glo H_2_O_2_ assay kit (Promega). Briefly, 1 × 10^5^ cells were cultured in 80 μl of medium on 96-well plates. Then, 20 μl of the H_2_O_2_ substrate solution was added to the cells and incubated for 2 h with or without 400 μM H_2_O_2_ treatment. Subsequently, 100 μl of ROS-Glo Detection Solution was added and incubated for 20 min at RT, followed by the measurement of relative luminescence units using a GloMax 96 Microplate Luminometer (Promega).

### *In vitro* 8-OHdG excision activity assay

The base excision activity colorimetric assay was performed as previously described with slight modification^37^,^38^. This assay is based on an HRP-mimicking DNAzyme consisting of a guanine quadruplex sequence and lambda exonuclease. Endogenous DNA glycosylases excise the 8-OHdG site and produce a 5′ phosphate and a 3′-phospho-unsaturated aldehyde in annealed oligonucleotides. Then, lambda exonuclease digests the 5′-phosphoryl strand of the annealed DNA to release the complementary DNAzyme sequence, which acts as an HRP, catalyzing the H_2_O_2_-mediated oxidation of 2,2′-azino-bis-(3-ethylbenzthiazoline-6-sulfonic acid) (ABTS^2−^, Abcam) to generate a colorimetric signal. In this study, 10 μM of 8-OHdG including oligonucleotide (DNA-1), its complementary sequence containing the DNAzyme part (DNA-2) and 5 μl NEBuffer2 (New England BioLabs, Tokyo, Japan) was denatured for 5 min at 90 °C, followed by annealing with slow cooling to RT. As a negative control, DNA-3 was used in place of DNA-1. For this experiment, 6 × 10^6^ cells on 10 cm dishes were harvested and incubated in 150 μl of extraction buffer (50 mM Tris–HCl pH 7.5, 150 mM NaCl, 0.05% NP-40, 2 mM EDTA, 0.1% PMFS) for 30 min at 4 °C, followed by centrifugation at 20,000*g* for 30 min. Then, 10 μl of the supernatant lysates, 10 μg ml^−1^ BSA and 5 units of lambda exonuclease were mixed with the annealed oligo and incubated for 40 min at 37 °C to cleave the 8-OHdG site and digest the DNA-1 strand. Subsequently, 15 μl of 5 μM hemin, 75 μl of 2 × HEPES solution (25 mM HEPES, 200 mM NaCl, 10 mM KCl, 0.05% triton, pH 5.2) and 10 μl water were added and incubated for 30 min at RT. Absorption spectra were recorded at 410 nm every 1 min after the addition of 20 μl of 20 mM ABTS^2−^ and 20 μl of 10 mM H_2_O_2_. The oligonucleotides sequences were as follows: DNA-1: 5′-TCTCG*ATCCCAACCCGCCCTACCC-3′ (*modified 8-OHdG); DNA-2: 5′-GGGTAGGGCGGGTTGGGATCGAGA-3′; DNA-3: 5′-TCTCGATCCCAACCCGCCCTACCC-3′.

### MNU-HPRT colony formation assay

K512-vector, K512-MajSAT and K512-MajSAT-YBX1-GFP cells were treated for 1 week with hypoxanthine–aminopterin–thymidine (HAT) medium (Sigma) to eliminate pre-existing mutated cells in the HPRT gene. To promote the opportunities for acquiring oxidation-induced mutations, MNU (Wako) was added for 1 week or 2 weeks. For this experiment, 6 × 10^6^ cells were plated on each 10 cm dish with 5 μM 6-TG (Wako)-containing media, which is catalyzed to cell-toxic 6-mercaptopurine by functioning HPRT. After 20 days, surviving colonies, with potential mutations in the HPRT gene, were counted by crystal violet staining.

### mtDNA copy number measurement

Total DNA was isolated from 1 × 10^6^ cells using the QIAamp DNA mini kit (Qiagen) according to the manufacturer's protocol. Then, 100 ng of template was subjected to quantitative SYBR green PCR (StepOnePlus Realtime PCR system; Invitrogen). Each sample was run in triplicate. The average threshold cycle number (Ct) values of mtDNA (mt-Co1 and mt-Cytb) and nuclear DNA (H19) were obtained, and the relative content of mtDNA, normalized by the Ct values of nuclear-H19, was calculated using the delta-delta Ct (ddCt) method. The primers used were as follows^70^: mt-Co1, Fw: 5′-CCCAATCTCTACCAGCATC-3′ and Rv: 5′-GGCTCATAGTATAGCTGGAG-3′; mt-Cytb, Fw: 5′-TTCTGAGGTGCCACAGTTATT-3′ and Rv: 5′-GAAGGAAAGGTATTAGGGCTAAA-3′; and nuclear-H19, Fw: 5′-GTACCCACCTGTCGTCC-3′ and Rv: 5′-GTCCACGAGACCAATGACTG-3′.

### Determination of the YBX1-binding domain

To construct serial YBX1 deletion mutants (A, B and C), YBX1 coding sequences with various deletions were subcloned into the pLVSIN-EF1α-neo vector at the XbaI and BamHI sites using the InFusion HD cloning kit, with a flag tag added to the 5′ end. The pLVSIN-EF1α-YBX1GFP vector was used as a template, and the primers used were as follows: Fw: 5′- GGATTTAAATTCTAGAACCATGGACTACAAGGATG-3′; mutant YBX1-A Rv: 5′-CGGTAGAATTGGATCCTTACCCGCCGGCGGGCGC-3′; mutant YBX1-B Rv: 5′-CGGTAGAATTGGATCCTTATGTAACATTTGCTGCCTC-3′; and mutant YBX1-C Rv: 5′-CGGTAGAATTGGATCCTTACTCCATCACTTCTCCTT-3′.

To examine the interaction between the mutant YBX1 protein and MajSAT RNA, RNA immunoprecipitation was performed. Briefly, 1 × 10^6^ 293TN cells were seeded in a 10 cm dish, transfected with 2 μg of the flag-tagged YBX1-overexpression mutant vector or the control vector, and incubated for 48 h. Cells were harvested, lysed and incubated with precleared Protein G agarose beads for 1 h. The supernatants were mixed with 2 μg MajSAT-Fw RNA, which was synthesized by *in vitro* transcription, and agarose beads conjugated to anti-flag antibody or control IgG for 3 h at 4 °C. The pellets were washed four times, and bound RNA was isolated by ethanol precipitation and resuspended in 10 μl RNase-free water. Precipitation of flag-tagged protein was confirmed by western blotting. To detect bound MajSAT RNA, 6 μl precipitated RNA and 5% input were reverse transcribed into cDNA using SuperScript III Reverse Transcriptase (Invitrogen), and semi-quantitative RT-PCR was performed as described earlier. Precipitated samples that were not reverse transcribed were used as a negative control.

### Statistical analysis

Statistically significant differences between groups were identified using Student's *t* test when the variances were equal. When the variances were unequal, Welch's *t* test was used instead. *P* values <0.05 were considered to indicate statistical significance.

### Data availability

The exome sequence data have been deposited in the Sequence Read Archive database (SRA, http://www.ncbi.nlm.nih.gov/sra) under the accession code #SRP081008'. All the other data supporting the findings of this study are available within the article and its Supplementary Information and from the corresponding author upon reasonable request.

## Additional information

**How to cite this article:** Kishikawa, T. *et al.* Satellite RNAs promote pancreatic oncogenic processes via the dysfunction of YBX1. *Nat. Commun.* **7,** 13006 doi: 10.1038/ncomms13006 (2016).

## Supplementary Material

Supplementary Information Supplementary Figures 1-11 and Supplementary Table 1

Supplementary Data 1 Single nucleotide variances in K512-TREtight-MajSAT cells.

Supplementary Data 2 Small insertions and deletions in K512-TREtight-MajSAT cells.

## References

[b1] Bardeesy N. & DePinho R. A. Pancreatic cancer biology and genetics. Nat. Rev. Cancer 2, 897–909 (2002). 12459728 10.1038/nrc949

[b2] Hruban R. H., Goggins M., Parsons J. & Kern S. E. Progression model for pancreatic cancer. Clin. Cancer Res. 6, 2969–2972 (2000). 10955772

[b3] Biankin A. V. *et al.* Pancreatic cancer genomes reveal aberrations in axon guidance pathway genes. Nature 491, 399–405 (2012). 23103869 10.1038/nature11547 PMC3530898

[b4] Waddell N. *et al.* Whole genomes redefine the mutational landscape of pancreatic cancer. Nature 518, 495–501 (2015). 25719666 10.1038/nature14169 PMC4523082

[b5] Jones S. *et al.* Core signaling pathways in human pancreatic cancers revealed by global genomic analyses. Science 321, 1801–1806 (2008). 18772397 10.1126/science.1164368 PMC2848990

[b6] Knudsen E. S., O'Reilly E. M., Brody J. R. & Witkiewicz A. K. Genetic diversity of pancreatic ductal adenocarcinoma and opportunities for precision medicine. Gastroenterology 150, 48–63 (2016). 26385075 10.1053/j.gastro.2015.08.056 PMC5010785

[b7] Vogelstein B. & Kinzler K. W. The path to cancer—three strikes and you're out. N. Engl. J. Med. 373, 1895–1898 (2015). 26559569 10.1056/NEJMp1508811

[b8] Lohr M., Kloppel G., Maisonneuve P., Lowenfels A. B. & Luttges J. Frequency of K-ras mutations in pancreatic intraductal neoplasias associated with pancreatic ductal adenocarcinoma and chronic pancreatitis: a meta-analysis. Neoplasia 7, 17–23 (2005). 15720814 10.1593/neo.04445 PMC1490318

[b9] Ijichi H. *et al.* Aggressive pancreatic ductal adenocarcinoma in mice caused by pancreas-specific blockade of transforming growth factor-beta signaling in cooperation with active Kras expression. Genes Dev. 20, 3147–3160 (2006). 17114585 10.1101/gad.1475506 PMC1635149

[b10] Plohl M., Mestrovic N. & Mravinac B. Centromere identity from the DNA point of view. Chromosoma 123, 313–325 (2014). 24763964 10.1007/s00412-014-0462-0 PMC4107277

[b11] Biscotti M. A., Canapa A., Forconi M., Olmo E. & Barucca M. Transcription of tandemly repetitive DNA: functional roles. Chromosome Res. 23, 463–477 (2015). 26403245 10.1007/s10577-015-9494-4

[b12] Rosic S., Kohler F. & Erhardt S. Repetitive centromeric satellite RNA is essential for kinetochore formation and cell division. J. Cell Biol. 207, 335–349 (2014). 25365994 10.1083/jcb.201404097 PMC4226727

[b13] Ferri F., Bouzinba-Segard H., Velasco G., Hube F. & Francastel C. Non-coding murine centromeric transcripts associate with and potentiate Aurora B kinase. Nucleic Acids Res. 37, 5071–5080 (2009). 19542185 10.1093/nar/gkp529 PMC2731909

[b14] Ideue T., Cho Y., Nishimura K. & Tani T. Involvement of satellite I noncoding RNA in regulation of chromosome segregation. Genes Cells 19, 528–538 (2014). 24750444 10.1111/gtc.12149

[b15] Probst A. V. *et al.* A strand-specific burst in transcription of pericentric satellites is required for chromocenter formation and early mouse development. Dev. Cell 19, 625–638 (2010). 20951352 10.1016/j.devcel.2010.09.002

[b16] Jachowicz J. W., Santenard A., Bender A., Muller J. & Torres-Padilla M. E. Heterochromatin establishment at pericentromeres depends on nuclear position. Genes Dev. 27, 2427–2432 (2013). 24240232 10.1101/gad.224550.113 PMC3841731

[b17] Terranova R., Sauer S., Merkenschlager M. & Fisher A. G. The reorganisation of constitutive heterochromatin in differentiating muscle requires HDAC activity. Exp. Cell Res. 310, 344–356 (2005). 16182285 10.1016/j.yexcr.2005.07.031

[b18] Ting D. T. *et al.* Aberrant overexpression of satellite repeats in pancreatic and other epithelial cancers. Science 331, 593–596 (2011). 21233348 10.1126/science.1200801 PMC3701432

[b19] Zhu Q. *et al.* BRCA1 tumour suppression occurs via heterochromatin-mediated silencing. Nature 477, 179–184 (2011). 21901007 10.1038/nature10371 PMC3240576

[b20] Bersani F. *et al.* Pericentromeric satellite repeat expansions through RNA-derived DNA intermediates in cancer. Proc. Natl Acad. Sci. USA 112, 15148–15153 (2015). 26575630 10.1073/pnas.1518008112 PMC4679016

[b21] Lyabin D. N., Eliseeva I. A. & Ovchinnikov L. P. YB-1 protein: functions and regulation. Wiley Interdiscip. Rev. RNA 5, 95–110 (2014). 24217978 10.1002/wrna.1200

[b22] Eliseeva I. A., Kim E. R., Guryanov S. G., Ovchinnikov L. P. & Lyabin D. N. Y-box-binding protein 1 (YB-1) and its functions. Biochemistry (Mosc.) 76, 1402–1433 (2011). 22339596 10.1134/S0006297911130049

[b23] Goodarzi H. *et al.* Endogenous tRNA-derived fragments suppress breast cancer progression via YBX1 displacement. Cell 161, 790–802 (2015). 25957686 10.1016/j.cell.2015.02.053 PMC4457382

[b24] Das S. *et al.* Stimulation of NEIL2-mediated oxidized base excision repair via YB-1 interaction during oxidative stress. J. Biol. Chem. 282, 28474–28484 (2007). 17686777 10.1074/jbc.M704672200 PMC2679419

[b25] Koike K. *et al.* Nuclear translocation of the Y-box binding protein by ultraviolet irradiation. FEBS Lett. 417, 390–394 (1997). 9409758 10.1016/s0014-5793(97)01296-9

[b26] Bouzinba-Segard H., Guais A. & Francastel C. Accumulation of small murine minor satellite transcripts leads to impaired centromeric architecture and function. Proc. Natl Acad. Sci. USA 103, 8709–8714 (2006). 16731634 10.1073/pnas.0508006103 PMC1482643

[b27] Tagarro I., Fernandez-Peralta A. M. & Gonzalez-Aguilera J. J. Chromosomal localization of human satellites 2 and 3 by a FISH method using oligonucleotides as probes. Hum. Genet. 93, 383–388 (1994). 8168808 10.1007/BF00201662

[b28] Jolly C. *et al.* Stress-induced transcription of satellite III repeats. J. Cell Biol. 164, 25–33 (2004). 14699086 10.1083/jcb.200306104 PMC2171959

[b29] Valgardsdottir R. *et al.* Transcription of Satellite III non-coding RNAs is a general stress response in human cells. Nucleic Acids Res. 36, 423–434 (2008). 18039709 10.1093/nar/gkm1056 PMC2241877

[b30] Chatterjee A., Dasgupta S. & Sidransky D. Mitochondrial subversion in cancer. Cancer Prev. Res. 4, 638–654 (2011). 10.1158/1940-6207.CAPR-10-0326 PMC3298745 21543342

[b31] Khaidakov M., Heflich R. H., Manjanatha M. G., Myers M. B. & Aidoo A. Accumulation of point mutations in mitochondrial DNA of aging mice. Mutat. Res. 526, 1–7 (2003). 12714177 10.1016/s0027-5107(03)00010-1

[b32] Rinn J. L. & Chang H. Y. Genome regulation by long noncoding RNAs. Annu. Rev. Biochem. 81, 145–166 (2012). 22663078 10.1146/annurev-biochem-051410-092902 PMC3858397

[b33] Batista P. J., Chang H. Y. & Long Noncoding, RNAs: cellular address codes in development and disease. Cell 152, 1298–1307 (2013). 23498938 10.1016/j.cell.2013.02.012 PMC3651923

[b34] Pestryakov P. *et al.* Effect of the multifunctional proteins RPA, YB-1, and XPC repair factor on AP site cleavage by DNA glycosylase NEIL1. J. Mol. Recognit. 25, 224–233 (2012). 22434712 10.1002/jmr.2182

[b35] Jalal S., Earley J. N. & Turchi J. J. DNA repair: from genome maintenance to biomarker and therapeutic target. Clin. Cancer Res. 17, 6973–6984 (2011). 21908578 10.1158/1078-0432.CCR-11-0761 PMC3218201

[b36] Mitra S., Boldogh I., Izumi T. & Hazra T. K. Complexities of the DNA base excision repair pathway for repair of oxidative DNA damage. Environ. Mol. Mutagen. 38, 180–190 (2001). 11746753 10.1002/em.1070 PMC4927302

[b37] Liu S.-C., Wu H.-W., Jiang J.-h., Shen G.-L. & Yu R.-Q. A novel DNAzyme-based colorimetric assay for the detection of hOGG1 activity with lambda exonuclease cleavage. Anal. Methods 5, 164–168 (2013).

[b38] Chatterjee N., Eom H. J. & Choi J. Effects of silver nanoparticles on oxidative DNA damage-repair as a function of p38 MAPK status: a comparative approach using human Jurkat T cells and the nematode *Caenorhabditis elegans*. Environ. Mol. Mutagen. 55, 122–133 (2014). 24347047 10.1002/em.21844

[b39] Verschoor M. L. *et al.* Mitochondria and cancer: past, present, and future. Biomed. Res. Int. 2013, 1–10 (2013). 10.1155/2013/612369 PMC3581248 23509753

[b40] Leonova K. I. *et al.* p53 cooperates with DNA methylation and a suicidal interferon response to maintain epigenetic silencing of repeats and noncoding RNAs. Proc. Natl Acad. Sci. USA 110, E89–E98 (2013). 23236145 10.1073/pnas.1216922110 PMC3538199

[b41] Lehnertz B. *et al.* Suv39h-mediated histone H3 lysine 9 methylation directs DNA methylation to major satellite repeats at pericentric heterochromatin. Curr. Biol. 13, 1192–1200 (2003). 12867029 10.1016/s0960-9822(03)00432-9

[b42] Tilman G. *et al.* Cancer-linked satellite 2 DNA hypomethylation does not regulate Sat2 non-coding RNA expression and is initiated by heat shock pathway activation. Epigenetics 7, 903–913 (2012). 22722874 10.4161/epi.21107 PMC3427286

[b43] Eymery A., Callanan M. & Vourc'h C. The secret message of heterochromatin: new insights into the mechanisms and function of centromeric and pericentric repeat sequence transcription. Int. J. Dev. Biol. 53, 259–268 (2009). 19412885 10.1387/ijdb.082673ae

[b44] Millanes-Romero A. *et al.* Regulation of heterochromatin transcription by Snail1/LOXL2 during epithelial-to-mesenchymal transition. Mol. Cell 52, 746–757 (2013). 24239292 10.1016/j.molcel.2013.10.015

[b45] Bulut-Karslioglu A. *et al.* A transcription factor-based mechanism for mouse heterochromatin formation. Nat. Struct. Mol. Biol. 19, 1023–1030 (2012). 22983563 10.1038/nsmb.2382

[b46] Tanne A. *et al.* Distinguishing the immunostimulatory properties of noncoding RNAs expressed in cancer cells. Proc. Natl Acad. Sci. USA 112, 15154–15159 (2015). 26575629 10.1073/pnas.1517584112 PMC4679042

[b47] Ohsawa S. *et al.* Mitochondrial defect drives non-autonomous tumour progression through Hippo signalling in Drosophila. Nature 490, 547–551 (2012). 23023132 10.1038/nature11452

[b48] Brandon M., Baldi P. & Wallace D. C. Mitochondrial mutations in cancer. Oncogene 25, 4647–4662 (2006). 16892079 10.1038/sj.onc.1209607

[b49] Carew J. S. & Huang P. Mitochondrial defects in cancer. Mol. Cancer 1, 9 (2002). 12513701 10.1186/1476-4598-1-9 PMC149412

[b50] Modica-Napolitano J. S., Kulawiec M. & Singh K. K. Mitochondria and human cancer. Curr. Mol. Med. 7, 121–131 (2007). 17311537 10.2174/156652407779940495

[b51] Maison C. *et al.* SUMOylation promotes de novo targeting of HP1alpha to pericentric heterochromatin. Nat. Genet. 43, 220–227 (2011). 21317888 10.1038/ng.765

[b52] Ise T. *et al.* Transcription factor Y-box binding protein 1 binds preferentially to cisplatin-modified DNA and interacts with proliferating cell nuclear antigen. Cancer Res. 59, 342–346 (1999). 9927044

[b53] Gaudreault I., Guay D. & Lebel M. YB-1 promotes strand separation *in vitro* of duplex DNA containing either mispaired bases or cisplatin modifications, exhibits endonucleolytic activities and binds several DNA repair proteins. Nucleic Acids Res. 32, 316–327 (2004). 14718551 10.1093/nar/gkh170 PMC373280

[b54] En-Nia A. *et al.* Transcription factor YB-1 mediates DNA polymerase alpha gene expression. J. Biol. Chem. 280, 7702–7711 (2005). 15615704 10.1074/jbc.M413353200

[b55] Lasham A. *et al.* The Y-box-binding protein, YB1, is a potential negative regulator of the p53 tumor suppressor. J. Biol. Chem. 278, 35516–35523 (2003). 12835324 10.1074/jbc.M303920200

[b56] Guay D., Gaudreault I., Massip L. & Lebel M. Formation of a nuclear complex containing the p53 tumor suppressor, YB-1, and the Werner syndrome gene product in cells treated with UV light. Int. J. Biochem. Cell Biol. 38, 1300–1313 (2006). 16584908 10.1016/j.biocel.2006.01.008

[b57] Machado A. M. D. *et al.* Helicobacter pylori infection affects mitochondrial function and DNA repair, thus, mediating genetic instability in gastric cells. Mech. Ageing Dev. 134, 460–466 (2013). 24012633 10.1016/j.mad.2013.08.004

[b58] de Souza-Pinto N. C. *et al.* Novel DNA mismatch-repair activity involving YB-1 in human mitochondria. DNA Repair (Amst.) 8, 704–719 (2009). 19272840 10.1016/j.dnarep.2009.01.021 PMC2693314

[b59] Clay Montier L. L., Deng J. J. & Bai Y. Number matters: control of mammalian mitochondrial DNA copy number. J. Genet. Genomics 36, 125–131 (2009). 19302968 10.1016/S1673-8527(08)60099-5 PMC4706993

[b60] Chytil A., Magnuson M. A., Wright C. V. & Moses H. L. Conditional inactivation of the TGF-beta type II receptor using Cre:Lox. Genesis 32, 73–75 (2002). 11857781 10.1002/gene.10046

[b61] Kawaguchi Y. *et al.* The role of the transcriptional regulator Ptf1a in converting intestinal to pancreatic progenitors. Nat. Genet. 32, 128–134 (2002). 12185368 10.1038/ng959

[b62] Jackson E. L. *et al.* Analysis of lung tumor initiation and progression using conditional expression of oncogenic K-ras. Genes Dev. 15, 3243–3248 (2001). 11751630 10.1101/gad.943001 PMC312845

[b63] Ijichi H. *et al.* Inhibiting Cxcr2 disrupts tumor–stromal interactions and improves survival in a mouse model of pancreatic ductal adenocarcinoma. J. Clin. Invest. 121, 4106–4117 (2011). 21926469 10.1172/JCI42754 PMC3195452

[b64] Vo K. *et al.* Targeting notch pathway enhances rapamycin antitumor activity in pancreas cancers through PTEN phosphorylation. Mol. Cancer 10, 138 (2011). 22074495 10.1186/1476-4598-10-138 PMC3253061

[b65] Yoshikawa T. *et al.* Unique haploinsufficient role of the microRNA-processing molecule Dicer1 in a murine colitis-associated tumorigenesis model. PLoS ONE 8, e71969 (2013). 24023722 10.1371/journal.pone.0071969 PMC3759383

[b66] Koboldt D. C. *et al.* VarScan 2: somatic mutation and copy number alteration discovery in cancer by exome sequencing. Genome Res. 22, 568–576 (2012). 22300766 10.1101/gr.129684.111 PMC3290792

[b67] Li H. & Durbin R. Fast and accurate short read alignment with Burrows–Wheeler transform. Bioinformatics 25, 1754–1760 (2009). 19451168 10.1093/bioinformatics/btp324 PMC2705234

[b68] Cingolani P. *et al.* A program for annotating and predicting the effects of single nucleotide polymorphisms, SnpEff: SNPs in the genome of *Drosophila melanogaster* strain w1118; iso-2; iso-3. Fly (Austin) 6, 80–92 (2012). 22728672 10.4161/fly.19695 PMC3679285

[b69] Yoshikawa T. *et al.* Silencing of microRNA-122 enhances interferon-alpha signaling in the liver through regulating SOCS3 promoter methylation. Sci. Rep. 2, 637 (2012). 22957141 10.1038/srep00637 PMC3434395

[b70] Hayashi M. *et al.* A crucial role of mitochondrial Hsp40 in preventing dilated cardiomyopathy. Nat. Med. 12, 128–132 (2006). 16327803 10.1038/nm1327

