# Supplementary material for: Satellite RNAs promote pancreatic oncogenic processes via the dysfunction of YBX1
Source: Nat Commun. 2016 Sep 26;7:13006. doi: 10.1038/ncomms13006 (PMC5052683; doi:10.1038/ncomms13006)
Supplement: Supplementary Information — Supplementary Figures 1-11 and Supplementary Table 1 [file ncomms13006-s1.pdf]

Supplementary Figure 1

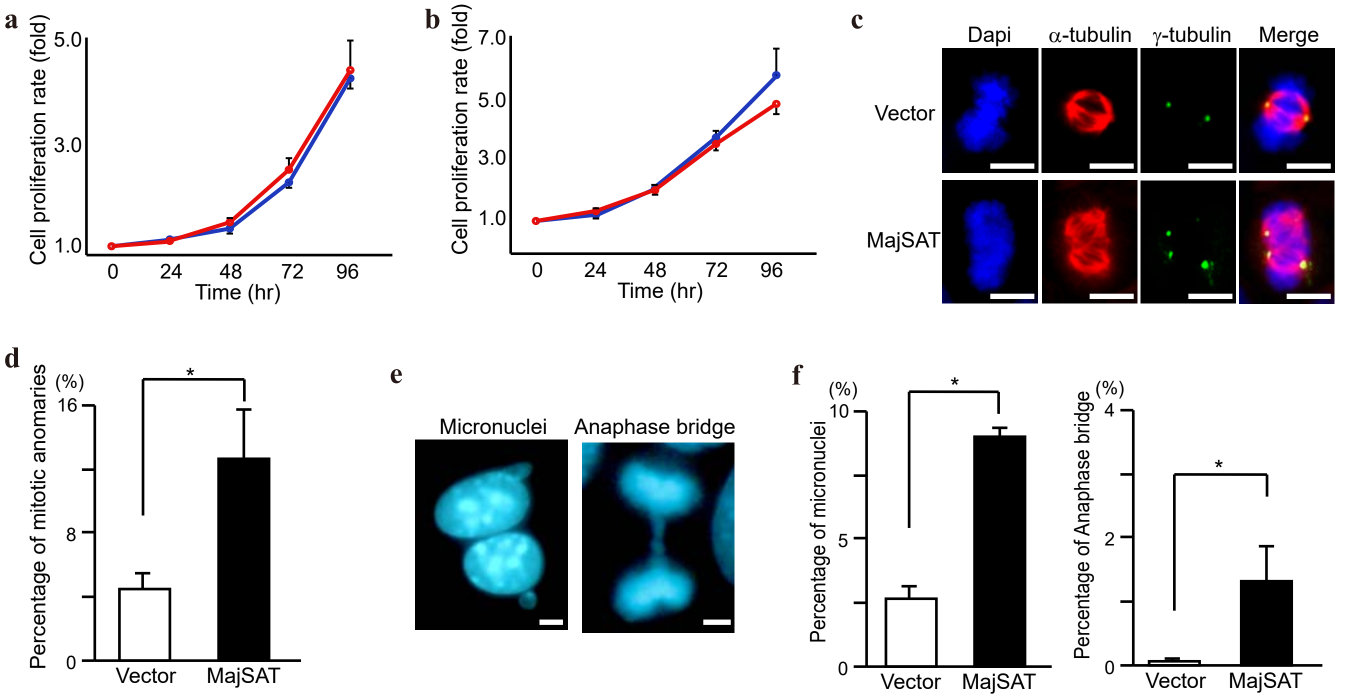

Supplementary Figure 1 | MajSAT expression causes chromosomal instability. a, b, The cell growth curve did not significantly change with MajSAT RNA expression. a, Blue: K512-vector; red: K512-EF1α-MajSAT. b, K512-TREtight-MajSAT cells. Blue: mock red: doxycycline treatment. Data represent the mean ± s.e. of four independent experiments. **c,** Representative images of multi-polar mitosis in K512-EF1α-MajSAT (MajSAT) cells. α-tubulin indicates microtubule. γ-tubulin indicates centrosome. **d,** Percentage of multi-polar mitotic cells in all mitotic cells in K512-EF1α-MajSAT (MajSAT) cells. Mitotic cells were counted, and the ratio of abnormal mitosis was determined out of 200 mitotic cells counted. Data represent the mean ± s.e. of four independent experiments. *: $p < 0.05$. **e,** Representative images of micronuclei (left) and anaphase bridge (right) in K512-EF1α-majSAT (MajSAT) cells. **f,** Percentage of interphase cells with micronuclei and anaphase bridge was calculated out of the 500 cells counted. Data represent the mean ± s.e. of four independent experiments. *: $p < 0.05$.

Supplementary Figure 2

```
Dloop-amplified seq   1 -------TCTTTTTATTTTGGCCTACTTTCATCAACATAGCCGTCAAGGCATGAAAGGACAGCACACAGTCTAGACGCACCTACGGTGAA 83
512L-dloop-c48-T7    84 TGGATCC................................................................................... 173
512L-dloop-c49-T7    86 TGGATCC................................................................................... 175
512L-dloop-c50-T7    85 TGGATCC................................................................................... 174
512L-dloop-c51-T7    86 TGGATCC................................................................................... 175
512L-dloop-c52-T7    86 TGGATCC................................................................................... 175
512L-dloop-c53-T7    84 TGGATCC................................................................................... 173
512L-dloop-c54-T7    85 TGGATCC................................................................................... 174
512L-dloop-c55-T7    85 TGGATCC................................................................................... 174
512L-dloop-c56-T7    84 TGGATCC................................................................................... 173
512L-dloop-c57-T7    84 TGGATCC................................................................................... 173
512L-dloop-c58-T7    85 TGGATCC................................................................................... 174
512L-dloop-c59-T7    85 TGGATCC................................................................................... 174
512L-dloop-c60-T7    85 TGGATCC.................................................................................G. 174
512L-dloop-c61-T7    85 TGGATCC................................................................................... 174
512L-dloop-c62-T7    85 TGGATCC................................................................................... 174
512L-dloop-c64-T7    84 TGGATCC................................................................................... 173
512L-dloop-c65-T7    85 TGGATCC................................................................................... 174
512L-dloop-c66-T7    84 TGGATCC................................................................................... 173
512L-dloop-c67-T7    84 TGGATCC................................................................................... 173
512L-dloop-c68-T7    85 TGGATCC................................................................................... 174
512L-dloop-c69-T7    85 TGGATCC................................................................................... 174
512L-dloop-c70-T7    85 TGGATCC................................................................................... 174
512L-dloop-c71-T7    87 TGGATCC................................................................................... 176
512L-dloop-c72-T7    86 TGGATCC................................................................................... 175
512L-dloop-c73-T7    84 TGGATCC................................................................................... 173
512L-dloop-c74-T7    85 TGGATCC................................................................................... 174
512L-dloop-c75-T7    84 TGGATCC................................................................................... 173
512L-dloop-c76-T7    84 TGGATCC................................................................................... 173
512L-dloop-c77-T7    86 TGGATCC................................................................................... 175
512L-dloop-c78-T7    86 TGGATCC................................................................................... 175
512L-dloop-c79-T7    84 TGGATCC................................................................................... 173
512L-dloop-c80-T7    85 TGGATCC................................................................................... 174
512L-dloop-c81-T7    84 TGGATCC................................................................................... 173
512L-dloop-c82-T7    84 TGGATCC................................................................................... 173
512L-dloop-c83-T7    84 TGGATCC................................................................................... 173
512L-dloop-c84-T7    84 TGGATCC................................................................................... 173
512L-dloop-c85-T7    85 TGGATCC................................................................................... 174
512L-dloop-c86-T7    85 TGGATCC................................................................................... 174
512L-dloop-c87-T7    87 TGGATCC................................................................................... 176
512L-dloop-c88-T7    85 TGGATCC................................................................................... 174
blank                19 ------------------------------------------------------------------------------------------ 19
512L-dloop-c1-T7     89 TGGATCC................................................................................... 178
512L-dloop-c2-T7     86 TGGATCC................................................................................... 175
512L-dloop-c3-T7     84 TGGATCC................................................................................... 173
512L-dloop-c4-T7     84 TGGATCC................................................................................... 173
512L-dloop-c5-T7     83 TGGATCC................................................................................... 172
512L-dloop-c6-T7     90 TGGATCC................................................................................... 179
512L-dloop-c8-T7     90 TGGATCC................................................................................... 179
512L-dloop-c9-T7     84 TGGATCC................................................................................... 173
512L-dloop-c10-T7    85 TGGATCC................................................................................... 174
512L-dloop-c11-T7    85 TGGATCC................................................................................... 174
512L-dloop-c13-T7    86 TGGATCC................................................................................... 175
512L-dloop-c14-T7    89 TGGATCC................................................................................... 178
512L-dloop-c15-T7    86 TGGATCC................................................................................... 175
512L-dloop-c16-T7    84 TGGATCC................................................................................... 173
512L-dloop-c17-T7    85 TGGATCC................................................................................... 174
512L-dloop-c18-T7    84 TGGATCC................................................................................... 173
512L-dloop-c20-T7    88 TGGATCC................................................................................... 177
512L-dloop-c21-T7    85 TGGATCC................................................................................... 174
512L-dloop-c22-T7    87 TGGATCC................................................................................... 176
512L-dloop-c23-T7    89 TGGATCC................................................................................... 178
512L-dloop-c24-T7    89 TGGATCC................................................................................... 178
```

```
Dloop-amplified seq  84 GAATCATTAGTCCGCAAAACCCAATCACCTAAGGCTAATTATTCATGCTTGTTAGACATAAATGCTACTCAATACCAAATTTTAACTCTC 173
512L-dloop-c48-T7   174 .......................................................................................... 263
512L-dloop-c49-T7   176 .......................................................................................... 265
512L-dloop-c50-T7   175 .......................................................................................... 264
512L-dloop-c51-T7   176 .......................................................................................... 265
512L-dloop-c52-T7   176 .......................................................................................... 265
512L-dloop-c53-T7   174 .......................................................................................... 263
512L-dloop-c54-T7   175 .......................................................................................... 264
512L-dloop-c55-T7   175 .......................................................................................... 264
512L-dloop-c56-T7   174 .......................................................................................... 263
512L-dloop-c57-T7   174 .......................................................................................... 263
512L-dloop-c58-T7   175 .......................................................................................... 264
512L-dloop-c59-T7   175 .......................................................................................... 264
512L-dloop-c60-T7   175 .......................................................................................... 264
512L-dloop-c61-T7   175 .......................................................................................... 264
512L-dloop-c62-T7   175 .......................................................................................... 264
512L-dloop-c64-T7   174 .......................................................................................... 263
512L-dloop-c65-T7   175 .......................................................................................... 264
512L-dloop-c66-T7   174 .......................................................................................... 263
512L-dloop-c67-T7   174 .......................................................................................... 263
512L-dloop-c68-T7   175 .......................................................................................... 264
512L-dloop-c69-T7   175 .......................................................................................... 264
512L-dloop-c70-T7   175 .......................................................................................... 264
512L-dloop-c71-T7   177 .......................................................................................... 266
512L-dloop-c72-T7   176 .......................................................................................... 265
512L-dloop-c73-T7   174 .......................................................................................... 263
512L-dloop-c74-T7   175 .......................................................................................... 264
512L-dloop-c75-T7   174 .......................................................................................... 263
512L-dloop-c76-T7   174 .......................................................................................... 263
512L-dloop-c77-T7   176 .......................................................................................... 265
512L-dloop-c78-T7   176 .......................................................................................... 265
512L-dloop-c79-T7   174 .......................................................................................... 263
512L-dloop-c80-T7   175 .......................................................................................... 264
512L-dloop-c81-T7   174 .......................................................................................... 263
512L-dloop-c82-T7   174 .......................................................................................... 263
512L-dloop-c83-T7   174 .......................................................................................... 263
512L-dloop-c84-T7   174 .......................................................................................... 263
512L-dloop-c85-T7   175 .......................................................................................... 264
512L-dloop-c86-T7   175 .......................................................................................... 264
512L-dloop-c87-T7   177 .......................................................................................... 266
512L-dloop-c88-T7   175 .......................................................................................... 264
blank                19 ------------------------------------------------------------------------------------------ 19
512L-dloop-c1-T7    179 .......................................................................................... 268
512L-dloop-c2-T7    176 .......................................................................................... 265
512L-dloop-c3-T7    174 .......................................................................................... 263
512L-dloop-c4-T7    174 .......................................................................................... 263
512L-dloop-c5-T7    173 .......................................................................................... 262
512L-dloop-c6-T7    180 .......................................................................................... 269
512L-dloop-c8-T7    180 .......................................................................................... 269
512L-dloop-c9-T7    174 ...................................................................................G...... 263
512L-dloop-c10-T7   175 .......................................................................................... 264
512L-dloop-c11-T7   175 .......................................................................................... 264
512L-dloop-c13-T7   176 .......................................................................................... 265
512L-dloop-c14-T7   179 .......................................................................................... 268
512L-dloop-c15-T7   176 .......................................................................................... 265
512L-dloop-c16-T7   174 .......................................................................................... 263
512L-dloop-c17-T7   175 .......................................................................................... 264
512L-dloop-c18-T7   174 .......................................................................................... 263
512L-dloop-c20-T7   178 .......................................................................................... 267
512L-dloop-c21-T7   175 .......................................................................................... 264
512L-dloop-c22-T7   177 .......................................................................................... 266
512L-dloop-c23-T7   179 .......................................................................................... 268
512L-dloop-c24-T7   179 .......................................................................................... 268
```

```
Dloop-amplified seq  174 CAAACCCCCCACCCCCTCCTCTTAATGCCAAACCCCAAAAACACTAAGAACTTGAAAGACATATAATATTAACTATCAAACCCTATGTCC 263
512L-dloop-c48-T7    264 .......................................................................................... 353
512L-dloop-c49-T7    266 .......................................................................................... 355
512L-dloop-c50-T7    265 .......................................................................................... 354
512L-dloop-c51-T7    266 .......................................................................................... 355
512L-dloop-c52-T7    266 .......................................................................................... 355
512L-dloop-c53-T7    264 .......................................................................................... 353
512L-dloop-c54-T7    265 .......................................................................................... 354
512L-dloop-c55-T7    265 .......................................................................................... 354
512L-dloop-c56-T7    264 .......................................................................................... 353
512L-dloop-c57-T7    264 .......................................................................................... 353
512L-dloop-c58-T7    265 .......................................................................................... 354
512L-dloop-c59-T7    265 .......................................................................................... 354
512L-dloop-c60-T7    265 .......................................................................................... 354
512L-dloop-c61-T7    265 .......................................................................................... 354
512L-dloop-c62-T7    265 .......................................................................................... 354
512L-dloop-c64-T7    264 .......................................................................................... 353
512L-dloop-c65-T7    265 .......................................................................................... 354
512L-dloop-c66-T7    264 .......................................................................................... 353
512L-dloop-c67-T7    264 .......................................................................................... 353
512L-dloop-c68-T7    265 .......................................................................................... 354
512L-dloop-c69-T7    265 .......................................................................................... 354
512L-dloop-c70-T7    265 .......................................................................................... 354
512L-dloop-c71-T7    267 .......................................................................................... 356
512L-dloop-c72-T7    266 .......................................................................................... 355
512L-dloop-c73-T7    264 .......................................................................................... 353
512L-dloop-c74-T7    265 .......................................................................................... 354
512L-dloop-c75-T7    264 .......................................................................................... 353
512L-dloop-c76-T7    264 .......................................................................................... 353
512L-dloop-c77-T7    266 .......................................................................................... 355
512L-dloop-c78-T7    266 .......................................................................................... 355
512L-dloop-c79-T7    264 .......................................................................................... 353
512L-dloop-c80-T7    265 .......................................................................................... 354
512L-dloop-c81-T7    264 .......................................................................................... 353
512L-dloop-c82-T7    264 .......................................................................................... 353
512L-dloop-c83-T7    264 .......................................................................................... 353
512L-dloop-c84-T7    264 .......................................................................................... 353
512L-dloop-c85-T7    265 .......................................................................................... 354
512L-dloop-c86-T7    265 .......................................................................................... 354
512L-dloop-c87-T7    267 .......................................................................................... 356
512L-dloop-c88-T7    265 .......................................................................................... 354
blank                 19 ------------------------------------------------------------------------------------------ 19
512L-dloop-c1-T7     269 .......................................................................................... 358
512L-dloop-c2-T7     266 .......................................................................................... 355
512L-dloop-c3-T7     264 .......................................................................................... 353
512L-dloop-c4-T7     264 .......................................................................................... 353
512L-dloop-c5-T7     263 .......................................................................................... 352
512L-dloop-c6-T7     270 .......................................................................................... 359
512L-dloop-c8-T7     270 .......................................................................................... 359
512L-dloop-c9-T7     264 .......................................................................................... 353
512L-dloop-c10-T7    265 .......................................................................................... 354
512L-dloop-c11-T7    265 .......................................................................................... 354
512L-dloop-c13-T7    266 .......................................................................................... 355
512L-dloop-c14-T7    269 .......................................................................................... 358
512L-dloop-c15-T7    266 .......................................................................................... 355
512L-dloop-c16-T7    264 .......................................................................................... 353
512L-dloop-c17-T7    265 .........................C................................................................ 354
512L-dloop-c18-T7    264 .......................................................................................... 353
512L-dloop-c20-T7    268 .......................................................................................... 357
512L-dloop-c21-T7    265 .......................................................................................... 354
512L-dloop-c22-T7    267 .......................................................................................... 356
512L-dloop-c23-T7    269 .......................................................................................... 358
512L-dloop-c24-T7    269 .......................................................................................... 358
```

```
Dloop-amplified seq  264 TGATCAATTCTAGTAGTTCCCAAAATATGACTTATATTTTAGTACTTGTAAAAATTTTACAAAATCATGTTCCGTGAACCAAAACTCTAA 353
512L-dloop-c48-T7    354 .......................................................................................... 443
512L-dloop-c49-T7    356 .......................................................................................... 445
512L-dloop-c50-T7    355 .......................................................................................... 444
512L-dloop-c51-T7    356 .......................................................................................... 445
512L-dloop-c52-T7    356 .......................................................................................... 445
512L-dloop-c53-T7    354 .......................................................................................... 443
512L-dloop-c54-T7    355 .......................................................................................... 444
512L-dloop-c55-T7    355 .......................................................................................... 444
512L-dloop-c56-T7    354 .......................................................................................... 443
512L-dloop-c57-T7    354 .......................................................................................... 443
512L-dloop-c58-T7    355 .......................................................................................... 444
512L-dloop-c59-T7    355 .......................................................................................... 444
512L-dloop-c60-T7    355 .......................................................................................... 444
512L-dloop-c61-T7    355 ..............................................C........................................... 444
512L-dloop-c62-T7    355 .......................................................................................... 444
512L-dloop-c64-T7    354 .......................................................................................... 443
512L-dloop-c65-T7    355 .......................................................................................... 444
512L-dloop-c66-T7    354 .......................................................................................... 443
512L-dloop-c67-T7    354 .......................................................................................... 443
512L-dloop-c68-T7    355 .......................................................................................... 444
512L-dloop-c69-T7    355 .......................................................................................... 444
512L-dloop-c70-T7    355 .......................................................................................... 444
512L-dloop-c71-T7    357 .......................................................................................... 446
512L-dloop-c72-T7    356 .......................................................................................... 445
512L-dloop-c73-T7    354 .......................................................................................... 443
512L-dloop-c74-T7    355 .......................................................................................... 444
512L-dloop-c75-T7    354 .......................................................................................... 443
512L-dloop-c76-T7    354 .......................................................................................... 443
512L-dloop-c77-T7    356 .......................................................................................... 445
512L-dloop-c78-T7    356 .......................................................................................... 445
512L-dloop-c79-T7    354 .......................................................................................... 443
512L-dloop-c80-T7    355 .......................................................................................... 444
512L-dloop-c81-T7    354 .......................................................................................... 443
512L-dloop-c82-T7    354 .......................................................................................... 443
512L-dloop-c83-T7    354 .......................................................................................... 443
512L-dloop-c84-T7    354 .......................................................................................... 443
512L-dloop-c85-T7    355 .......................................................................................... 444
512L-dloop-c86-T7    355 .......................................................................................... 444
512L-dloop-c87-T7    357 .......................................................................................... 446
512L-dloop-c88-T7    355 .......................................................................................... 444
blank                 19 ------------------------------------------------------------------------------------------ 19
512L-dloop-c1-T7     359 .......................................................................................... 448
512L-dloop-c2-T7     356 .......................................................................................... 445
512L-dloop-c3-T7     354 .......................................................................................... 443
512L-dloop-c4-T7     354 .......................................................................................... 443
512L-dloop-c5-T7     353 .......................................................................................... 442
512L-dloop-c6-T7     360 .......................................................................................... 449
512L-dloop-c8-T7     360 .......................................................................................... 449
512L-dloop-c9-T7     354 .......................................................................................... 443
512L-dloop-c10-T7    355 .......................................................................................... 444
512L-dloop-c11-T7    355 .......................................................................................... 444
512L-dloop-c13-T7    356 .......................................................................................... 445
512L-dloop-c14-T7    359 .......................................................................................... 448
512L-dloop-c15-T7    356 .......................................................................................... 445
512L-dloop-c16-T7    354 .......................................................................................... 443
512L-dloop-c17-T7    355 .......................................................................................... 444
512L-dloop-c18-T7    354 .......................................................................................... 443
512L-dloop-c20-T7    358 .......................................................................................... 447
512L-dloop-c21-T7    355 .......................................................................................... 444
512L-dloop-c22-T7    357 .......................................................................................... 446
512L-dloop-c23-T7    359 .......................................................................................... 448
512L-dloop-c24-T7    359 ..........................................C............................................... 448
```

```
Dloop-amplified seq  354 TCATACTCTATTACGCAATAAACATTAACAAGTTAATGTAGCTTAATAACAAAGCAAAGCACTGAAAATGCTTAGATG------------ 431
512L-dloop-c48-T7    444 ..............................................................................GGATCCGAGCTC 533
512L-dloop-c49-T7    446 ..............................................................................GGATCCGAGCTC 535
512L-dloop-c50-T7    445 ..............................................................................GGATCCGAGCTC 534
512L-dloop-c51-T7    446 ..............................................................................GGATCCGAGCTC 535
512L-dloop-c52-T7    446 ..............................................................................GGATCCGAGCTC 535
512L-dloop-c53-T7    444 ..............................................................................GGATCCGAGCTC 533
512L-dloop-c54-T7    445 ..............................................................................GGATCCGAGCTC 534
512L-dloop-c55-T7    445 ..............................................................................GGATCCGAGCTC 534
512L-dloop-c56-T7    444 ..............................................................................GGATCCGAGCTC 533
512L-dloop-c57-T7    444 ..............................................................................GGATCCGAGCTC 533
512L-dloop-c58-T7    445 ..............................................................................GGATCCGAGCTC 534
512L-dloop-c59-T7    445 ..............................................................................GGATCCGAGCTC 534
512L-dloop-c60-T7    445 ..............................................................................GGATCCGAGCTC 534
512L-dloop-c61-T7    445 ..............................................................................GGATCCGAGCTC 534
512L-dloop-c62-T7    445 ..............................................................................GGATCCGAGCTC 534
512L-dloop-c64-T7    444 ..............................................................................GGATCCGAGCTC 533
512L-dloop-c65-T7    445 ..............................................................................GGATCCGAGCTC 534
512L-dloop-c66-T7    444 ..............................................................................GGATCCGAGCTC 533
512L-dloop-c67-T7    444 ..............................................................................GGATCCGAGCTC 533
512L-dloop-c68-T7    445 ..............................................................................GGATCCGAGCTC 534
512L-dloop-c69-T7    445 ..............................................................................GGATCCGAGCTC 534
512L-dloop-c70-T7    445 ..............................................................................GGATCCGAGCTC 534
512L-dloop-c71-T7    447 ..............................................................................GGATCCGAGCTC 536
512L-dloop-c72-T7    446 ..............................................................................GGATCCGAGCTC 535
512L-dloop-c73-T7    444 ..............................................................................GGATCCGAGCTC 533
512L-dloop-c74-T7    445 ..............................................................................GGATCCGAGCTC 534
512L-dloop-c75-T7    444 ..............................................................................GGATCCGAGCTC 533
512L-dloop-c76-T7    444 ..............................................................................GGATCCGAGCTC 533
512L-dloop-c77-T7    446 ..............................................................................GGATCCGAGCTC 535
512L-dloop-c78-T7    446 ..............................................................................GGATCCGAGCTC 535
512L-dloop-c79-T7    444 ..............................................................................GGATCCGAGCTC 533
512L-dloop-c80-T7    445 ..............................................................................GGATCCGAGCTC 534
512L-dloop-c81-T7    444 ..............................................................................GGATCCGAGCTC 533
512L-dloop-c82-T7    444 ..............................................................................GGATCCGAGCTC 533
512L-dloop-c83-T7    444 ..............................................................................GGATCCGAGCTC 533
512L-dloop-c84-T7    444 ..............................................................................GGATCCGAGCTC 533
512L-dloop-c85-T7    445 ..............................................................................GGATCCGAGCTC 534
512L-dloop-c86-T7    445 ..............................................................................GGATCCGAGCTC 534
512L-dloop-c87-T7    447 ..............................................................................GGATCCGAGCTC 536
512L-dloop-c88-T7    445 ..............................................................................GGATCCGAGCTC 534
blank                 19 ------------------------------------------------------------------------------------------ 19
512L-dloop-c1-T7     449 ..............................................................................GGATCCGAGCTC 538
512L-dloop-c2-T7     446 ..............................................................................GGATCCGAGCTC 535
512L-dloop-c3-T7     444 ..............................................................................GGATCCGAGCTC 533
512L-dloop-c4-T7     444 ..............................................................................GGATCCGAGCTC 533
512L-dloop-c5-T7     443 ..............................................................................GGATCCGAGCTC 532
512L-dloop-c6-T7     450 ..............................................................................GGATCCGAGCTC 539
512L-dloop-c8-T7     450 ..............................................................................GGATCCGAGCTC 539
512L-dloop-c9-T7     444 ..............................................................................GGATCCGAGCTC 533
512L-dloop-c10-T7    445 ..............................................................................GGATCCGAGCTC 534
512L-dloop-c11-T7    445 ..............................................................................GGATCCGAGCTC 534
512L-dloop-c13-T7    446 ..............................................................................GGATCCGAGCTC 535
512L-dloop-c14-T7    449 ..............................................................................GGATCCGAGCTC 538
512L-dloop-c15-T7    446 ..............................................................................GGATCCGAGCTC 535
512L-dloop-c16-T7    444 ..............................................................................GGATCCGAGCTC 533
512L-dloop-c17-T7    445 ..............................................................................GGATCCGAGCTC 534
512L-dloop-c18-T7    444 ..............................................................................GGATCCGAGCTC 533
512L-dloop-c20-T7    448 ..............................................................................GGATCCGAGCTC 537
512L-dloop-c21-T7    445 ..............................................................................GGATCCGAGCTC 534
512L-dloop-c22-T7    447 ..............................................................................GGATCCGAGCTC 536
512L-dloop-c23-T7    449 ..............................................................................GGATCCGAGCTC 538
512L-dloop-c24-T7    449 ..............................................................................GGATCCGAGCTC 538
```

Supplementary Figure 2 | Multiple alignment of the sequences in the D-loop region of mitochondrial DNA in K512-vector cells. Sequences in the D-loop region from control cells (K512-vector) are shown. The top line is the reference sequences: mouse consensus mitochondrial DNA (GenBank NC_005089.1, bases 15916-16299; 1-48). Mutated bases are indicated with frames.

Supplementary Figure 3

```
Dloop-amplified          1 ----------------TCTTTTTATTTTGGCCTACTTTCATCAACATAGCCGTCAAGGCATGAAAGGACAGCACACAGTC 64
512LM-dloop-c1-T7       77 CTGGACTAGTGGATCC................................................................ 156
512LM-dloop-c2-T7       78 CTGGACTAGTGGATCC................................................................ 157
512LM-dloop-c3-T7       80 CTGGACTAGTGGATCC................................................................ 159
512LM-dloop-c5-T7       78 CTGGACTAGTGGATCC................................................................ 157
512LM-dloop-c6-T7       79 CTGGACTAGTGGATCC................................................................ 158
512LM-dloop-c8-T7       77 CTGGACTAGTGGATCC................................................................ 156
512LM-dloop-c11-T7      78 CTGGACTAGTGGATCC................................................................ 157
512LM-dloop-c12-T7      77 CTGGACTAGTGGATCC................................................................ 156
512LM-dloop-c13-T7      76 CTGGACTAGTGGATCC................................................................ 155
512LM-dloop-c14-T7      75 CTGGACTAGTGGATCC................................................................ 154
512LM-dloop-c15-T7      75 CTGGACTAGTGGATCC................................................................ 154
512LM-dloop-c16-T7      76 CTGGACTAGTGGATCC................................................................ 155
512LM-dloop-c17-T7      76 CTGGACTAGTGGATCC................................................................ 155
blank                    5 -------------------------------------------------------------------------------- 5
512LM-dloop-c54-T7      75 CTGGACTAGTGGATCC................................................................ 154
512LM-dloop-c55-T7      76 CTGGACTAGTGGATCC................................................................ 155
512LM-dloop-c56-T7      75 CTGGACTAGTGGATCC...........A.................................................... 154
512LM-dloop-c57-T7      75 CTGGACTAGTGGATCC................................................................ 154
512LM-dloop-c58-T7      77 CTGGACTAGTGGATCC................................................................ 156
512LM-dloop-c59-T7      77 CTGGACTAGTGGATCC................................................................ 156
512LM-dloop-c60-T7      75 CTGGACTAGTGGATCC................................................................ 154
512LM-dloop-c61-T7      77 CTGGACTAGTGGATCC................................................................ 156
512LM-dloop-c62-T7      76 CTGGACTAGTGGATCC................................................................ 155
512LM-dloop-c63-T7      76 CTGGACTAGTGGATCC................................................................ 155
512LM-dloop-c64-T7      75 CTGGACTAGTGGATCC................................................................ 154
512LM-dloop-c65-T7      78 CTGGACTAGTGGATCC................................................................ 157
512LM-dloop-c66-T7      78 CTGGACTAGTGGATCC................................................................ 157
512LM-dloop-c67-T7      76 CTGGACTAGTGGATCC................................................................ 155
512LM-dloop-c68-T7      75 CTGGACTAGTGGATCC................................................................ 154
512LM-dloop-c69-T7      77 CTGGACTAGTGGATCC................................................................ 156
512LM-dloop-c70-T7      76 CTGGACTAGTGGATCC................................................................ 155
512LM-dloop-c71-T7      76 CTGGACTAGTGGATCC................................................................ 155
512LM-dloop-c72-T7      81 CTGGACTAGTGGATCC................................................................ 160
512LM-dloop-c73-T7      75 CTGGACTAGTGGATCC................................................................ 154
512LM-dloop-c74-T7      75 CTGGACTAGTGGATCC................................................................ 154
512LM-dloop-c75-T7      77 CTGGACTAGTGGATCC................................................................ 156
512LM-dloop-c76-T7      78 CTGGACTAGTGGATCC................................................................ 157
512LM-dloop-c77-T7      77 CTGGACTAGTGGATCC................................................................ 156
512LM-dloop-c78-T7      77 CTGGACTAGTGGATCC................................................................ 156
512LM-dloop-c79-T7      77 CTGGACTAGTGGATCC................................................................ 156
512LM-dloop-c80-T7      81 CTGGACTAGTGGATCC................................................................ 160
512LM-dloop-c81-T7      76 CTGGACTAGTGGATCC................................................................ 155
512LM-dloop-c82-T7      75 CTGGACTAGTGGATCC................................................................ 154
512LM-dloop-c83-T7      77 CTGGACTAGTGGATCC............A................................................... 156
512LM-dloop-c84-T7      77 CTGGACTAGTGGATCC................................................................ 156
512LM-dloop-c85-T7      77 CTGGACTAGTGGATCC................................................................ 156
512LM-dloop-c86-T7      77 CTGGACTAGTGGATCC................................................................ 156
512LM-dloop-c87-T7      75 CTGGACTAGTGGATCC................................................................ 154
512LM-dloop-c88-T7      76 CTGGACTAGTGGATCC................................................................ 155
512LM-dloop-c89-T7      79 CTGGACTAGTGGATCC................................................................ 158
512LM-dloop-c90-T7      77 CTGGACTAGTGGATCC................................................................ 156
512LM-dloop-c91-T7      79 CTGGACTAGTGGATCC................................................................ 158
512LM-dloop-c92-T7      78 CTGGACTAGTGGATCC................................................................ 157
512LM-dloop-c93-T7      75 CTGGACTAGTGGATCC................................................................ 154
512LM-dloop-c94-T7      78 CTGGACTAGTGGATCC................................................................ 157
```

```
Dloop-amplified      65  TAGACGCACCTACGGTGAAGAATCATTAGTCCGCAAAACCCAATCACCTAAGGCTAATTATTCATGCTTGTTAGACATAA  144
512LM-dloop-c1-T7   157  ................................................................................  236
512LM-dloop-c2-T7   158  ................................................................................  237
512LM-dloop-c3-T7   160  ................................................................................  239
512LM-dloop-c5-T7   158  ................................................................................  237
512LM-dloop-c6-T7   159  ................................................................................  238
512LM-dloop-c8-T7   157  ................................................................................  236
512LM-dloop-c11-T7  158  .............................C..................................................  237
512LM-dloop-c12-T7  157  ................................................................................  236
512LM-dloop-c13-T7  156  ................................................................................  235
512LM-dloop-c14-T7  155  ................................................................................  234
512LM-dloop-c15-T7  155  ................................................................................  234
512LM-dloop-c16-T7  156  ................................................................................  235
512LM-dloop-c17-T7  156  .....................................G..........................................  235
blank                 5  --------------------------------------------------------------------------------  5
512LM-dloop-c54-T7  155  ................................................................................  234
512LM-dloop-c55-T7  156  ................................................................................  235
512LM-dloop-c56-T7  155  ................................................................................  234
512LM-dloop-c57-T7  155  ................................................................................  234
512LM-dloop-c58-T7  157  ................................................................................  236
512LM-dloop-c59-T7  157  ................................................................................  236
512LM-dloop-c60-T7  155  ................................................................................  234
512LM-dloop-c61-T7  157  ................................................................................  236
512LM-dloop-c62-T7  156  ................................................................................  235
512LM-dloop-c63-T7  156  ................................................................................  235
512LM-dloop-c64-T7  155  ................................................................................  234
512LM-dloop-c65-T7  158  ................................................................................  237
512LM-dloop-c66-T7  158  ................................................................................  237
512LM-dloop-c67-T7  156  ................................................................................  235
512LM-dloop-c68-T7  155  ................................................................................  234
512LM-dloop-c69-T7  157  ................................................................................  236
512LM-dloop-c70-T7  156  ................................................................................  235
512LM-dloop-c71-T7  156  ................................................................................  235
512LM-dloop-c72-T7  161  ................................................................................  240
512LM-dloop-c73-T7  155  ............................................................................G...  234
512LM-dloop-c74-T7  155  ................................................................................  234
512LM-dloop-c75-T7  157  ................................................................................  236
512LM-dloop-c76-T7  158  ................................................................................  237
512LM-dloop-c77-T7  157  ................................................................................  236
512LM-dloop-c78-T7  157  ................................................................................  236
512LM-dloop-c79-T7  157  ................................................................................  236
512LM-dloop-c80-T7  161  ................................................................................  240
512LM-dloop-c81-T7  156  ................................................................................  235
512LM-dloop-c82-T7  155  ................................................................................  234
512LM-dloop-c83-T7  157  ................................................................................  236
512LM-dloop-c84-T7  157  ................................................................................  236
512LM-dloop-c85-T7  157  ................................................................................  236
512LM-dloop-c86-T7  157  ................................................................................  236
512LM-dloop-c87-T7  155  ................................................................................  234
512LM-dloop-c88-T7  156  ................................................................................  235
512LM-dloop-c89-T7  159  ................................................................................  238
512LM-dloop-c90-T7  157  ................................................................................  236
512LM-dloop-c91-T7  159  ................................................................................  238
512LM-dloop-c92-T7  158  ................................................................................  237
512LM-dloop-c93-T7  155  ................................................................................  234
512LM-dloop-c94-T7  158  ................................................................................  237
```

```
Dloop-amplified      145 ATGCTACTCAATACCAAATTTTAACTCTCCAAA-CCCCCCACCCCCTCCTCTTAATGCCAAACCCCAAAAACACTAAGAA 223
512LM-dloop-c1-T7    237 .................................-.............................................. 315
512LM-dloop-c2-T7    238 .................................-.............................................. 316
512LM-dloop-c3-T7    240 .................................-.............................................. 318
512LM-dloop-c5-T7    238 .................................-.............................................. 316
512LM-dloop-c6-T7    239 .................................-.............................................. 317
512LM-dloop-c8-T7    237 .................................-.............................................. 315
512LM-dloop-c11-T7   238 .................................-.............................................. 316
512LM-dloop-c12-T7   237 .................................-.............................................. 315
512LM-dloop-c13-T7   236 .................................-.............................................. 314
512LM-dloop-c14-T7   235 .................................-.............................................. 313
512LM-dloop-c15-T7   235 .................................-.............................................. 313
512LM-dloop-c16-T7   236 .................................-.............................................. 314
512LM-dloop-c17-T7   236 .................................-.............................................. 314
blank                  5 -------------------------------------------------------------------------------- 5
512LM-dloop-c54-T7   235 .................................-.............................................. 313
512LM-dloop-c55-T7   236 .................................-.............................................. 314
512LM-dloop-c56-T7   235 .................................-.............................................. 313
512LM-dloop-c57-T7   235 .................................-.............................................. 313
512LM-dloop-c58-T7   237 .................................-.............................................. 315
512LM-dloop-c59-T7   237 .................................-.............................................. 315
512LM-dloop-c60-T7   235 .................................-.............................................. 313
512LM-dloop-c61-T7   237 .................................-.............................................. 315
512LM-dloop-c62-T7   236 .................................-.............................................. 314
512LM-dloop-c63-T7   236 .................................-.............................................. 314
512LM-dloop-c64-T7   235 .................................-.............................................. 313
512LM-dloop-c65-T7   238 .................................-.............................................. 316
512LM-dloop-c66-T7   238 .................................-.............................................. 316
512LM-dloop-c67-T7   236 .................................-.............................................. 314
512LM-dloop-c68-T7   235 .................................-.............................................. 313
512LM-dloop-c69-T7   237 .................................-.............................................. 315
512LM-dloop-c70-T7   236 .................................-.............................................. 314
512LM-dloop-c71-T7   236 .................................-.............................................. 314
512LM-dloop-c72-T7   241 .................................-.............................................. 319
512LM-dloop-c73-T7   235 .................................-.............................................. 313
512LM-dloop-c74-T7   235 .................................-.............................................. 313
512LM-dloop-c75-T7   237 .................................-.............................................. 315
512LM-dloop-c76-T7   238 .................................-.............................................. 316
512LM-dloop-c77-T7   237 .................................-.............................................. 315
512LM-dloop-c78-T7   237 .................................-.............................................. 315
512LM-dloop-c79-T7   237 .................................C.............................................. 316
512LM-dloop-c80-T7   241 .................................-.............................................. 319
512LM-dloop-c81-T7   236 .................................-.............................................. 314
512LM-dloop-c82-T7   235 .................................-.............................................. 313
512LM-dloop-c83-T7   237 .................................-.............................................. 315
512LM-dloop-c84-T7   237 .................................-.............................................. 315
512LM-dloop-c85-T7   237 .................................-.............................................. 315
512LM-dloop-c86-T7   237 .................................-.............................................. 315
512LM-dloop-c87-T7   235 .................................-.............................................. 313
512LM-dloop-c88-T7   236 .................................-.............................................. 314
512LM-dloop-c89-T7   239 .................................-.............................................. 317
512LM-dloop-c90-T7   237 .................................-.............................................. 315
512LM-dloop-c91-T7   239 .................................-.............................................. 317
512LM-dloop-c92-T7   238 .................................-.............................................. 316
512LM-dloop-c93-T7   235 .................................-.............................................. 313
512LM-dloop-c94-T7   238 .................................-.............................................. 316
```

```
Dloop-amplified      224 CTTGAAAGACATATAATATTAACTATCAAACCCTATGTCCTGATCAATTCTAGTAGTTCCCAAAATATGACTTATATTTT 303
512LM-dloop-c1-T7    316 ................................................................................ 395
512LM-dloop-c2-T7    317 ................................................................................ 396
512LM-dloop-c3-T7    319 ................................................................................ 398
512LM-dloop-c5-T7    317 ................................................................................ 396
512LM-dloop-c6-T7    318 ................................................................................ 397
512LM-dloop-c8-T7    316 ................................................................................ 395
512LM-dloop-c11-T7   317 ................................................................................ 396
512LM-dloop-c12-T7   316 ................................................................................ 395
512LM-dloop-c13-T7   315 ................................................................................ 394
512LM-dloop-c14-T7   314 ................................................................................ 393
512LM-dloop-c15-T7   314 ................................................................................ 393
512LM-dloop-c16-T7   315 ................................................................................ 394
512LM-dloop-c17-T7   315 ................................................................................ 394
blank                  5 -------------------------------------------------------------------------------- 5
512LM-dloop-c54-T7   314 ................................................................................ 393
512LM-dloop-c55-T7   315 ................................................................................ 394
512LM-dloop-c56-T7   314 ................................................................................ 393
512LM-dloop-c57-T7   314 ................................................................................ 393
512LM-dloop-c58-T7   316 ................................................................................ 395
512LM-dloop-c59-T7   316 ................................................................................ 395
512LM-dloop-c60-T7   314 ................................................................................ 393
512LM-dloop-c61-T7   316 ................................................................................ 395
512LM-dloop-c62-T7   315 ................................................................................ 394
512LM-dloop-c63-T7   315 ................................................................................ 394
512LM-dloop-c64-T7   314 ................................................................................ 393
512LM-dloop-c65-T7   317 ................................................................................ 396
512LM-dloop-c66-T7   317 ................................................................................ 396
512LM-dloop-c67-T7   315 ................................................................................ 394
512LM-dloop-c68-T7   314 ................................................................................ 393
512LM-dloop-c69-T7   316 ................................................................................ 395
512LM-dloop-c70-T7   315 ................................................................................ 394
512LM-dloop-c71-T7   315 ................................................................................ 394
512LM-dloop-c72-T7   320 ................................................................................ 399
512LM-dloop-c73-T7   314 ................................................................................ 393
512LM-dloop-c74-T7   314 ................................................................................ 393
512LM-dloop-c75-T7   316 ................................................................................ 395
512LM-dloop-c76-T7   317 ................................................................................ 396
512LM-dloop-c77-T7   316 ................................................................................ 395
512LM-dloop-c78-T7   316 ................................................................................ 395
512LM-dloop-c79-T7   317 ................................................................................ 396
512LM-dloop-c80-T7   320 ................................................................................ 399
512LM-dloop-c81-T7   315 ................................................................................ 394
512LM-dloop-c82-T7   314 ................................................................................ 393
512LM-dloop-c83-T7   316 ................................................................................ 395
512LM-dloop-c84-T7   316 ................................................................................ 395
512LM-dloop-c85-T7   316 ................................................................................ 395
512LM-dloop-c86-T7   316 ................................................................................ 395
512LM-dloop-c87-T7   314 ................................................................................ 393
512LM-dloop-c88-T7   315 ................................................................................ 394
512LM-dloop-c89-T7   318 ................................................................................ 397
512LM-dloop-c90-T7   316 ................................................................................ 395
512LM-dloop-c91-T7   318 ................................................................................ 397
512LM-dloop-c92-T7   317 ................................................................................ 396
512LM-dloop-c93-T7   314 ................................................................................ 393
512LM-dloop-c94-T7   317 ................................................................................ 396
```

```
Dloop-amplified      304 AGTACTTGTAAAAATTTTACAAAATCATGTTCCGTGAACCAAAACTCTAATCATACTCTATTACGCAATAAACATTAACA 383
512LM-dloop-c1-T7    396 ................................................................................ 475
512LM-dloop-c2-T7    397 ................................................................................ 476
512LM-dloop-c3-T7    399 ................................................................................ 478
512LM-dloop-c5-T7    397 ................................................................................ 476
512LM-dloop-c6-T7    398 ................................................................................ 477
512LM-dloop-c8-T7    396 ................................................................................ 475
512LM-dloop-c11-T7   397 ................................................................................ 476
512LM-dloop-c12-T7   396 ................................................................................ 475
512LM-dloop-c13-T7   395 ................................................................................ 474
512LM-dloop-c14-T7   394 ................................................................................ 473
512LM-dloop-c15-T7   394 ................................................................................ 473
512LM-dloop-c16-T7   395 ................................................................................ 474
512LM-dloop-c17-T7   395 ................................................................................ 474
blank                  5 -------------------------------------------------------------------------------- 5
512LM-dloop-c54-T7   394 ................................................................................ 473
512LM-dloop-c55-T7   395 ................................................................................ 474
512LM-dloop-c56-T7   394 ................................................................................ 473
512LM-dloop-c57-T7   394 ................................................................................ 473
512LM-dloop-c58-T7   396 ................................................................................ 475
512LM-dloop-c59-T7   396 ...G............................................................................ 475
512LM-dloop-c60-T7   394 ................................................................................ 473
512LM-dloop-c61-T7   396 ................................................................................ 475
512LM-dloop-c62-T7   395 ................................................................................ 474
512LM-dloop-c63-T7   395 ................................................................................ 474
512LM-dloop-c64-T7   394 ................................................................................ 473
512LM-dloop-c65-T7   397 ................................................................................ 476
512LM-dloop-c66-T7   397 ................................................................................ 476
512LM-dloop-c67-T7   395 ................................................................................ 474
512LM-dloop-c68-T7   394 ................................................................................ 473
512LM-dloop-c69-T7   396 ................................................................................ 475
512LM-dloop-c70-T7   395 ................................................................................ 474
512LM-dloop-c71-T7   395 ................................................................................ 474
512LM-dloop-c72-T7   400 ................................................................................ 479
512LM-dloop-c73-T7   394 ................................................................................ 473
512LM-dloop-c74-T7   394 ................................................................................ 473
512LM-dloop-c75-T7   396 ................................................................................ 475
512LM-dloop-c76-T7   397 ................................................................................ 476
512LM-dloop-c77-T7   396 ................................................................................ 475
512LM-dloop-c78-T7   396 ................................................................................ 475
512LM-dloop-c79-T7   397 ................................................................................ 476
512LM-dloop-c80-T7   400 ................................................................................ 479
512LM-dloop-c81-T7   395 ................................................................................ 474
512LM-dloop-c82-T7   394 ................................................................................ 473
512LM-dloop-c83-T7   396 ................................................................................ 475
512LM-dloop-c84-T7   396 ................................................................................ 475
512LM-dloop-c85-T7   396 ................................................................................ 475
512LM-dloop-c86-T7   396 ................................................................................ 475
512LM-dloop-c87-T7   394 ................................................................................ 473
512LM-dloop-c88-T7   395 ................................................................................ 474
512LM-dloop-c89-T7   398 ................................................................................ 477
512LM-dloop-c90-T7   396 ................................................................................ 475
512LM-dloop-c91-T7   398 ................................................................................ 477
512LM-dloop-c92-T7   397 ................................................................................ 476
512LM-dloop-c93-T7   394 ................................................................................ 473
512LM-dloop-c94-T7   397 ................................................................................ 476
```

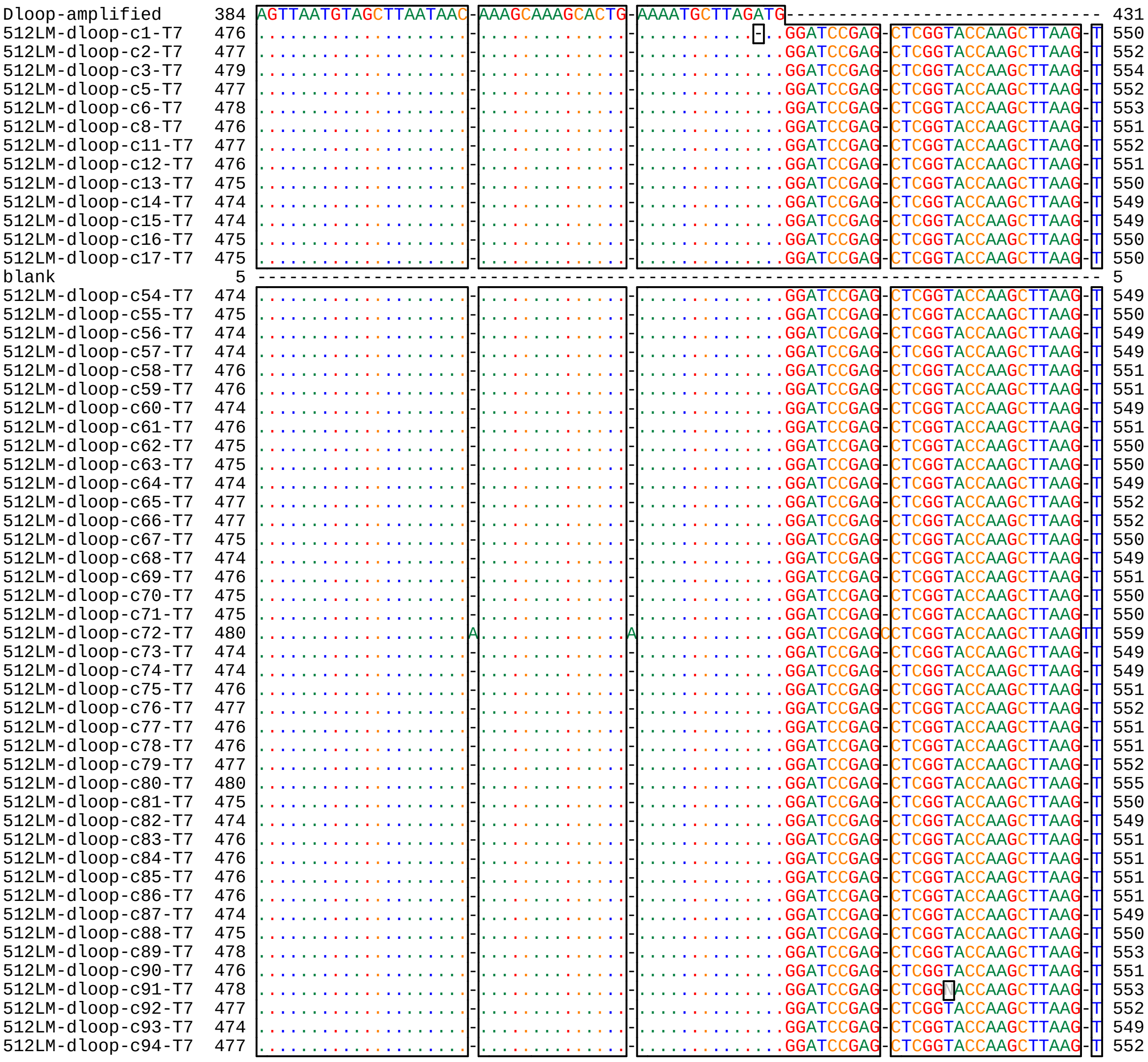

Supplementary Figure 3 | Multiple alignment of the sequences in the D-loop region of mitochondrial DNA in K512-EF1α-MajSAT cells. Sequences in the D-loop region from K512-EF1α-MajSAT cells are shown. The top line is the reference sequences. Mutated bases are surrounded with frames. Deleted bases are indicated by “-”.

Supplementary Figure 4

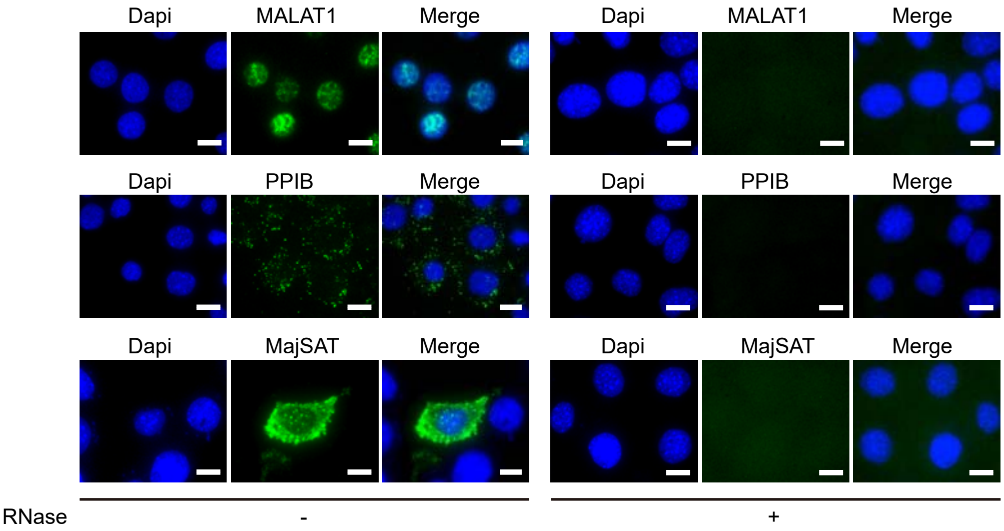

Supplementary Figure 4 | MajSAT RNA is distributed primarily in the cytoplasm. K512 cells were transiently transfected with pLVSIN-EF1α-MajSAT plasmid. MajSAT RNA was detected by in situ hybridization. MALAT1 RNA, a control for nuclear localization, and PPIB RNA, a control for cytoplasmic localization, were also evaluated. RNase-treated cells served as a negative control. Representative images from three independent experiments are shown. Bar: 10 µm.

Supplementary Figure 5

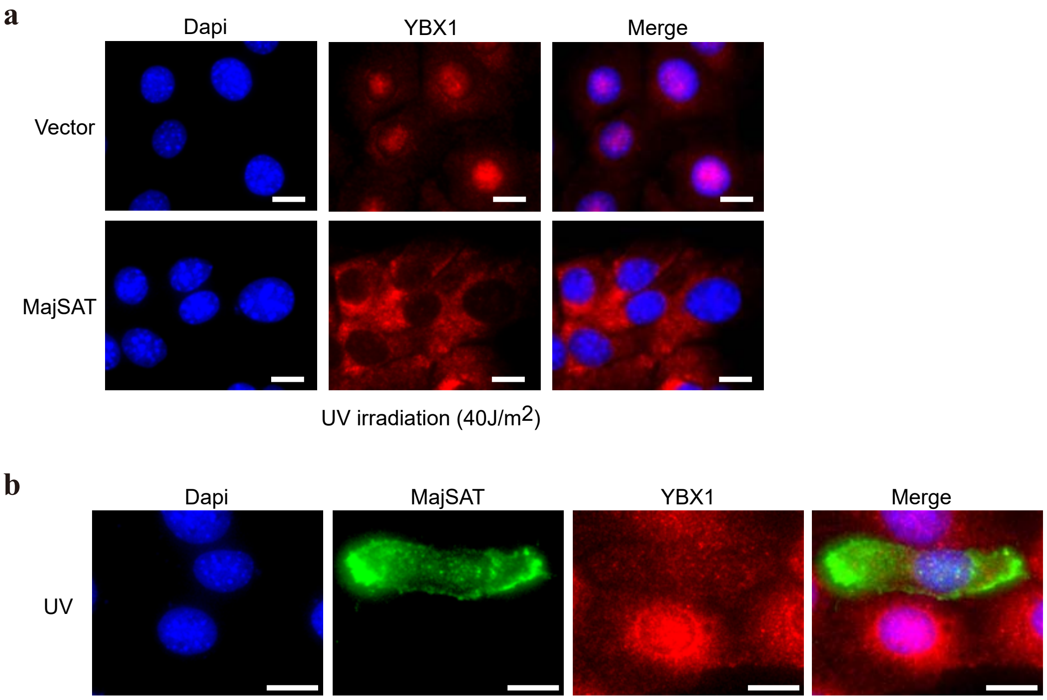

Supplementary Figure 5 | Nuclear translocation of YBX1 is impaired in MajSAT RNA-expressing cells after UV irradiation. a, YBX1 translocated into the nucleus after 40 J/m^2 UV irradiation in K512 cells with control vector, whereas in stably MajSAT RNA-expressing cells, YBX1 remained in the cytoplasm. Representative cell images of three independent experiments are shown. Bar: 10 µm. **b,** MajSAT RNA co-localized with YBX1 and inhibited nuclear transportation in UV irradiated cells. K512 cells transiently transfected with pLVSIN-EF1 α-MajSAT plasmid were irradiated at 40 J/m^2 UV. After 6 h, MajSAT RNA and YBX1 were double stained by in situ hybridization and immuno-fluorescence staining. A MajSAT RNA-expressing cell and untransfected cells are intentionally shown in the same field of view for comparison. Representative cell images from three independent experiments are shown. Bar: 10 µm.

Supplementary Figure 6

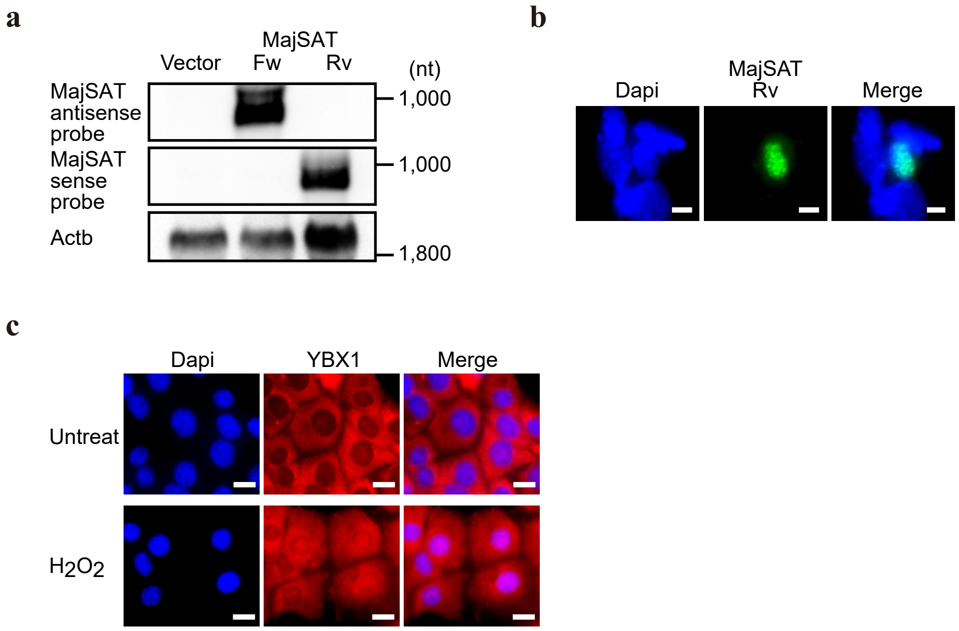

Supplementary Figure 6 | Cytoplasmic retention of YBX1 does not occur after expression of antisense MajSAT RNA. a, Antisense MajSAT RNA expression. K512 cells transiently transfected with sense MajSAT RNA (MajSAT Fw) or antisense MajSAT RNA (MajSAT Rv) expression constructs were used for northern blotting. The membrane was probed with sense and antisense MajSAT RNA probes and then re-probed with β-actin (Actb). nt, nucleotides. Representative results from three independent experiments are shown. **b,** Antisense MajSAT RNA was expressed primarily in the nucleus. K512 cells were transiently transfected with pLVSIN-EF1 α-MajSAT-Rv plasmid. Antisense MajSAT RNA was detected by in situ hybridization. Representative images from five independent experiments are shown. Bar: 10 µm. **c,** YBX1 was translocated into the nucleus in antisense MajSAT RNA-expressing cells following H_2O_2 stimulation. K512 cells stably expressing antisense MajSAT RNA were treated with or without 300 µM H_2O_2 for 6 h, and YBX1 immunostaining was performed. Representative images from five independent experiments are shown. Bar: 10 µm.

Supplementary Figure 7

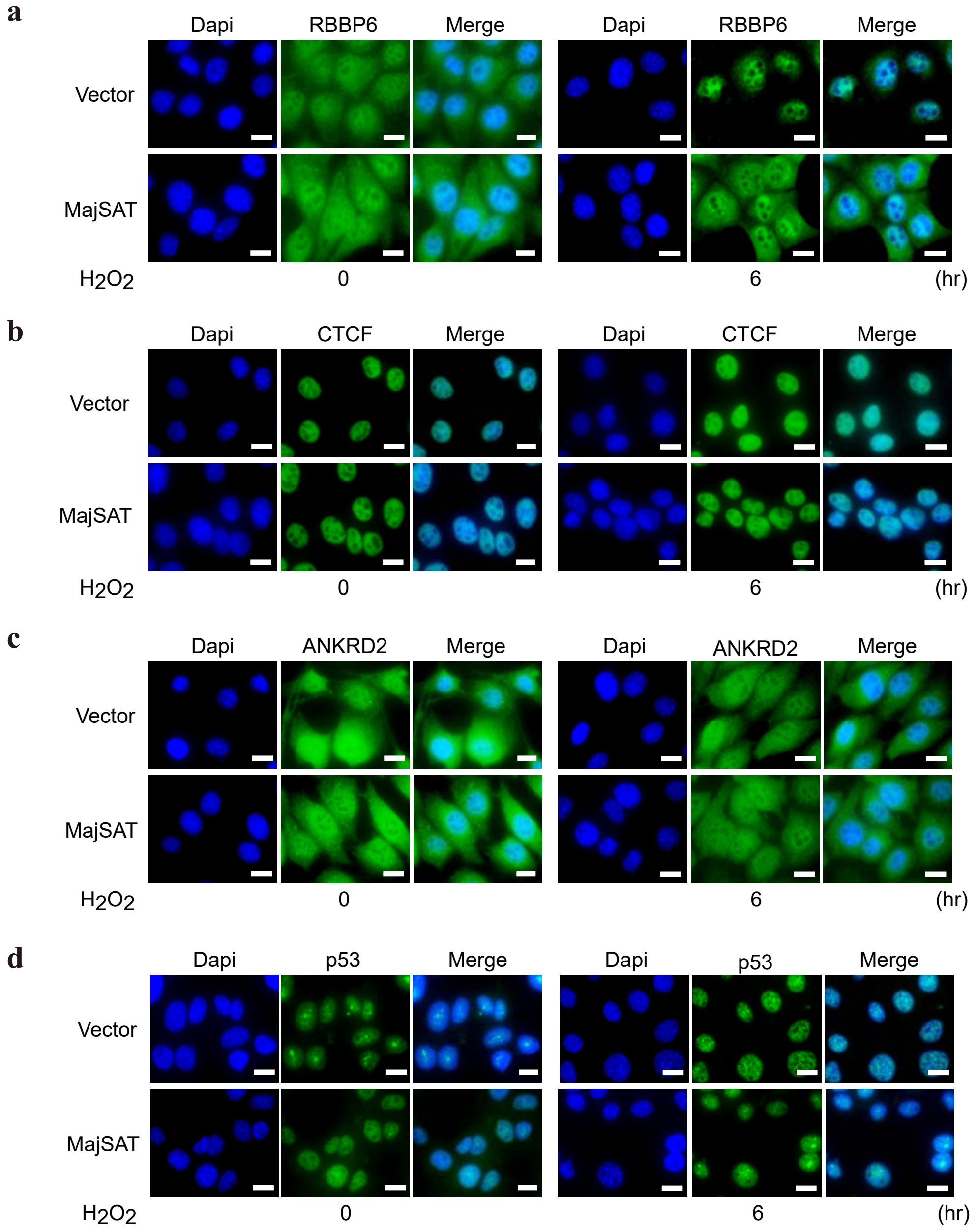

Supplementary Figure 7 | Intracellular localization of YBX1-interacting proteins in MajSAT RNA-expressing cells. K512 control (vector) and MajSAT RNA stably expressing K512 cells (MajSAT) were treated with or without 300 μM H_2O_2 for 6 h. YBX1-interacting proteins, RBBP6 (**a**), CTCF1 (**b**), ANKRD2 (**c**), and p53 (**d**), were subjected to immunostaining to visualize their intracellular localization. Translocation of RBBP6 into the nucleus following H_2O_2 treatment was partially impaired in MajSAT RNA-expressing cells. Representative images from three independent experiments are shown. Bar: 10 μm.

Supplementary Figure 8

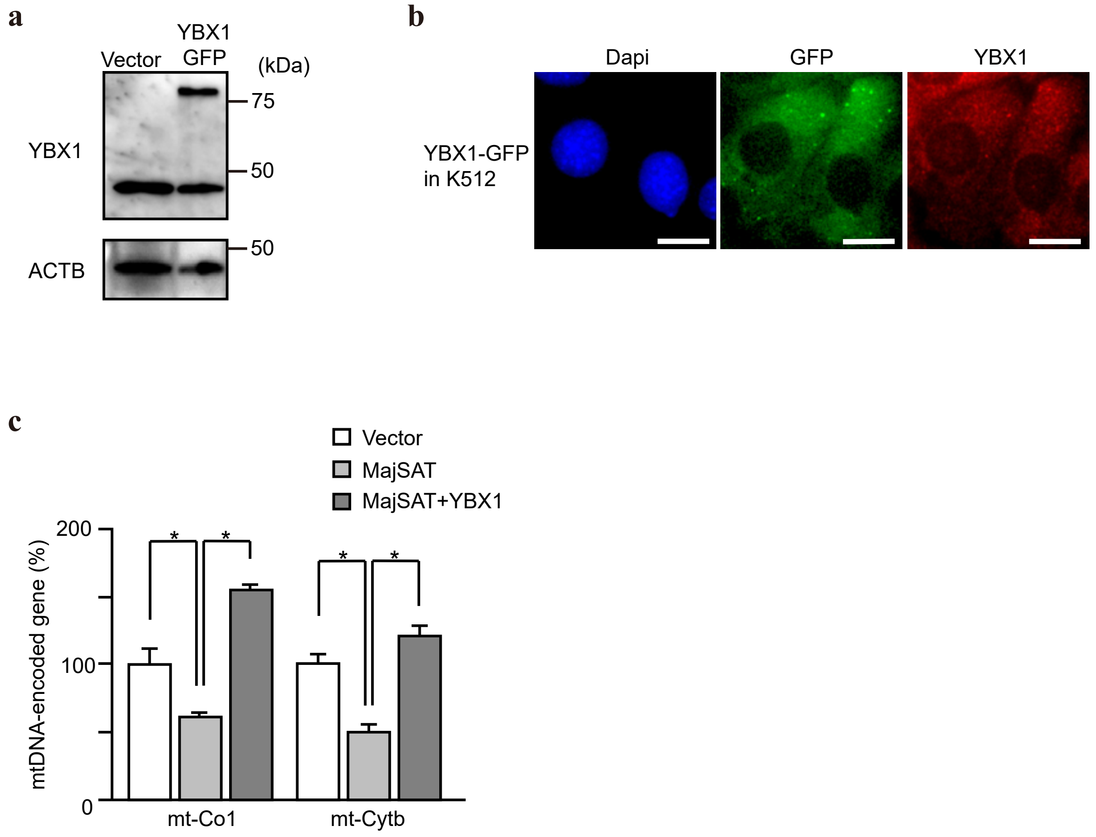

Supplementary Figure 8 | Forced YBX1 expression rescues MajSAT RNA-induced impaired function of YBX1. a, Western blotting of K512 cells stably overexpressing YBX1-GFP chimeric protein. The upper band (approximately 78 kDa) represents ectopic YBX1-GFP and the lower bands (approximately 48 kDa) represent endogenous YBX1 protein. Representative results of two independent experiments are shown. **b,** Immunofluorescent images of K512 stably expressing YBX1-GFP cells. YBX1 antibody reacts with both endogenous YBX1 and ectopic YBX-GFP. Representative cell images are shown. Bar: 10 µm. **c,** Mitochondria encoded genes (mt-Co1 and mt-Cytb) in K512 cells were quantified by quantitative PCR. The levels reflect mitochondrial DNA damages. Values from control cells were set as 100, and the values from the cells with MajSAT RNA expression with and without forced YBX1 expression were determined. Data represent the mean ± s.e. of four independent experiments. *: $p < 0.05$.

Supplementary Figure 9

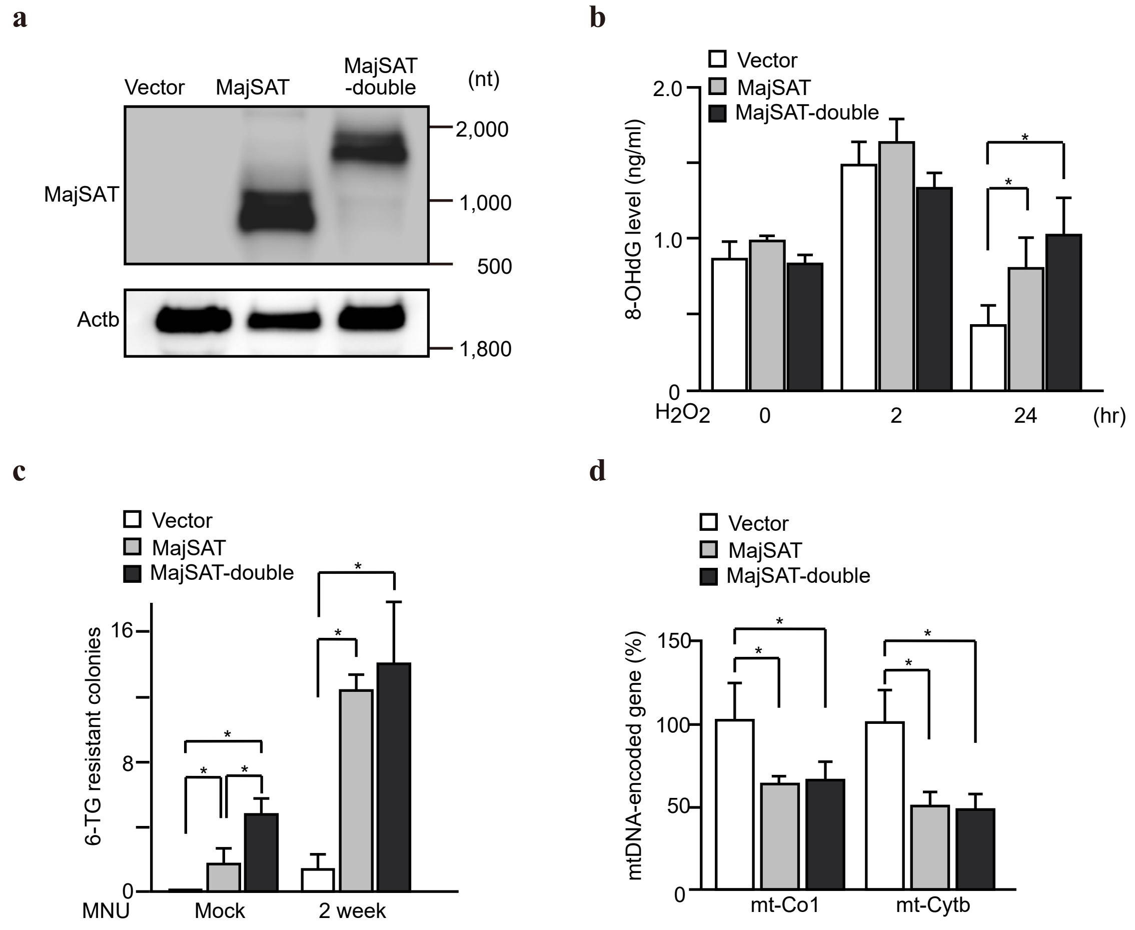

Supplementary Figure 9 | MajSAT RNA containing six basic units exhibits similar effects as those of MajSAT RNA containing three basic units. **a,** MajSAT RNA expression in K512 cells stably transduced with constitutively expressing constructs. MajSAT contains three basic units, while MajSAT-double contains longer sequences of six basic units. The same membrane was re-probed with β-actin (Actb). Nt: nucleotides. **b,** Recovery from oxidative DNA damage was delayed in a similar manner in MajSAT RNA-expressing cells (MajSAT) and MajSAT-double RNA-expressing cells (MajSAT-double). Levels of 8-OHdG were determined by competitive ELISA following H_2O_2 treatment for the indicated periods. Data represent the mean ± s.e. of three independent experiments. *, $p < 0.05$. **c,** Spontaneous missense mutations in the HPRT gene were increased in MajSAT-double cells similarly as MajSAT cells. Cells were treated with 2.0 μM MNU for 2 weeks to promote the tendency for mutation and subsequently cultured in 6-TG-containing media. The number of surviving colonies, which presumably developed mutations in the HPRT gene, was counted. Data represent the mean ± s.e. of three independent experiments. *, $p < 0.05$. **d,** Mitochondrial damage was similarly induced in MajSAT and MajSAT-double cells. Mitochondrially encoded genes (mt-Co1 and mt-Cytb) in K512 cells were quantified by quantitative PCR. The levels reflect mitochondrial DNA damage. Values from the control cells (vector) were set a 100%. Data represent the mean ± s.e. of four independent experiments. *, $p < 0.05$.

Supplementary Figure 10

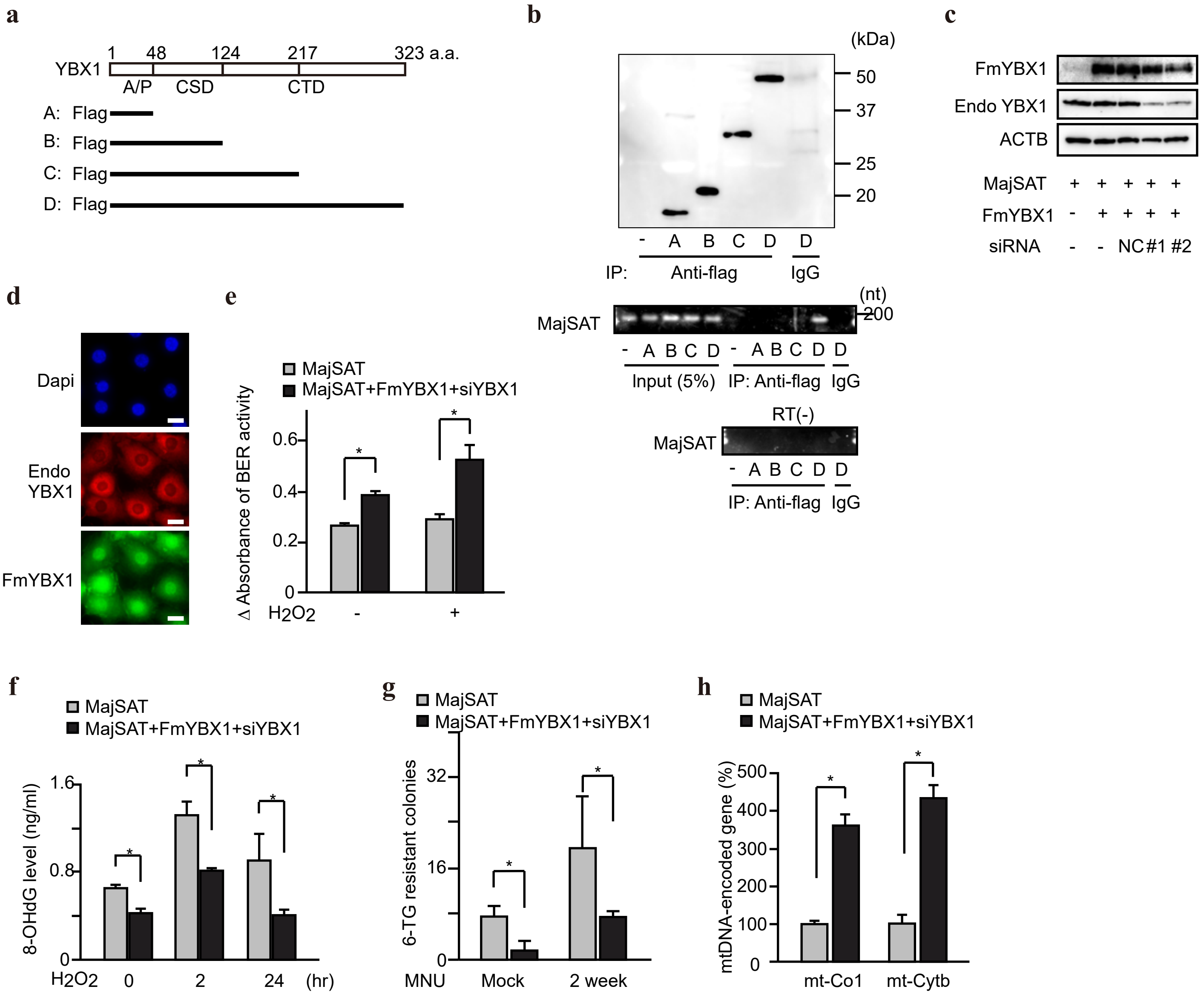

Supplementary Figure 10 | YBX1 that no longer interacts with MajSAT RNA is functional in MajSAT RNA-expressing cells. a, Deletion constructs of the YBX1 protein. Plasmids expressing C terminal domain-deleted and flag-tagged YBX1 were constructed as indicated (constructs A, B, C, and D). A/P: alanine and proline domain, CSD: cold shock domain, CTD: C-terminal domain, aa: amino acids. **b,** (Upper panel) 293TN cells were transiently transfected with flag-tagged YBX1-expressing plasmids as indicated, and cell lysates containing synthesized MajSAT RNA were subjected to immunoprecipitation (IP) with anti-flag antibodies. IP using isotype IgG with construct D was performed as a negative control. (Lower panels) The binding of MajSAT RNA to flag-tagged YBX1 proteins was determined by RT-PCR. Semi-quantitative RT-PCR images of MajSAT RNA are shown. Five percent of the cell lysate was used as a control (input). As a negative control, samples that were not subjected to reverse transcription were used and presented in the lower panel (RT(-)). Representative results from two independent experiments are shown. **c,** Replacement of endogenous YBX1 with the flag-tagged C-terminally deleted YBX1 (FmYBX1) that no longer interacts with MajSAT RNA. K512 cells stably expressing MajSAT RNA and FmYBX1 protein were transfected with siRNAs targeting the 5' untranslated region (#1) and exon 7 of YBX1 (deleted in construct C) (#2) to knock down endogenous YBX1 protein expression. NC: negative control siRNA. Protein levels of endogenous and mutant YBX1 were determined by western blotting using anti-C terminal YBX1 (recognizing only endogenous YBX1) and anti-flag antibodies (recognizing only mutant YBX1). The membrane was also probed with β-actin (ACTB) to confirm approximately equal protein loading. Representative results from two independent experiments are shown. **d,** FmYBX1 protein can translocate to the nucleus following H_2O_2 treatment. K512 cells stably expressing MajSAT RNA and FmYBX1 protein were treated with 300 μM H_2O_2 for 6 h. YBX1 was immunostained with the anti-flag antibody (green), which recognizes FmYBX1. Representative images from three independent experiments are shown. Bar: 10 μm. **e,** BER activity was recovered by replacing wild-type YBX1 with FmYBX1 in MajSAT RNA-expressing and endogenous YBX1 knock down cells (MajSAT + FmYBX1 + siYBX1). 8-OHdG excision activity was determined using a colorimetric DNAzyme assay. To determine the response to oxidative stress, cells were incubated in 400 μM H_2O_2 for 4 h. siRNA #1 in (c) was used for siYBX1. Data represent the mean ± s.e. of two independent experiments. *, $p < 0.05$. **f,** The recovery of oxidative DNA damage was restored by replacing wild-type YBX1 with FmYBX1 in MajSAT RNA-expressing and endogenous YBX1 knock down cells (MajSAT + FmYBX1 + siYBX1). Levels of 8-OHdG following H_2O_2 treatment for the indicated periods were determined by competitive ELISA. Data represent the mean ± s.e. of three independent experiments. *, $p < 0.05$. **g,** Spontaneous missense mutations in the HPRT gene were decreased in MajSAT + FmYBX1 + siYBX1 cells. Cells were treated with 2.0 μM MNU for 2 weeks to promote the tendency for mutation and subsequently cultured in 6-TG-containing media. The number of surviving colonies, which presumably developed mutations in the HPRT gene, was counted. Data represent the mean ± s.e. of three independent experiments. *, $p < 0.05$. **h,** Mitochondrial damage was recovered in MajSAT + FmYBX1 + siYBX1 cells. The expression of mitochondrially encoded genes (mt-Co1 and mt-Cytb), whose levels reflect mitochondrial damage, was quantified in K512 cells by quantitative PCR. Values from cells expressing MajSAT RNA only (MajSAT) were set at 100%. Data represent the mean ± s.e. of four independent experiments. *, $p < 0.05$.

Supplementary Figure 11

Figure 2b

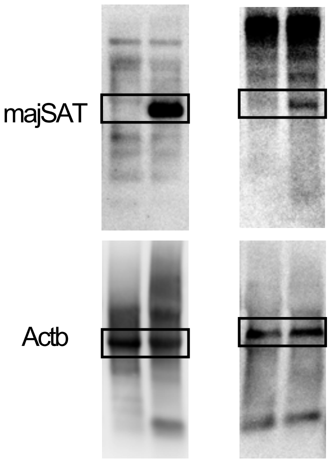

Figure 3b

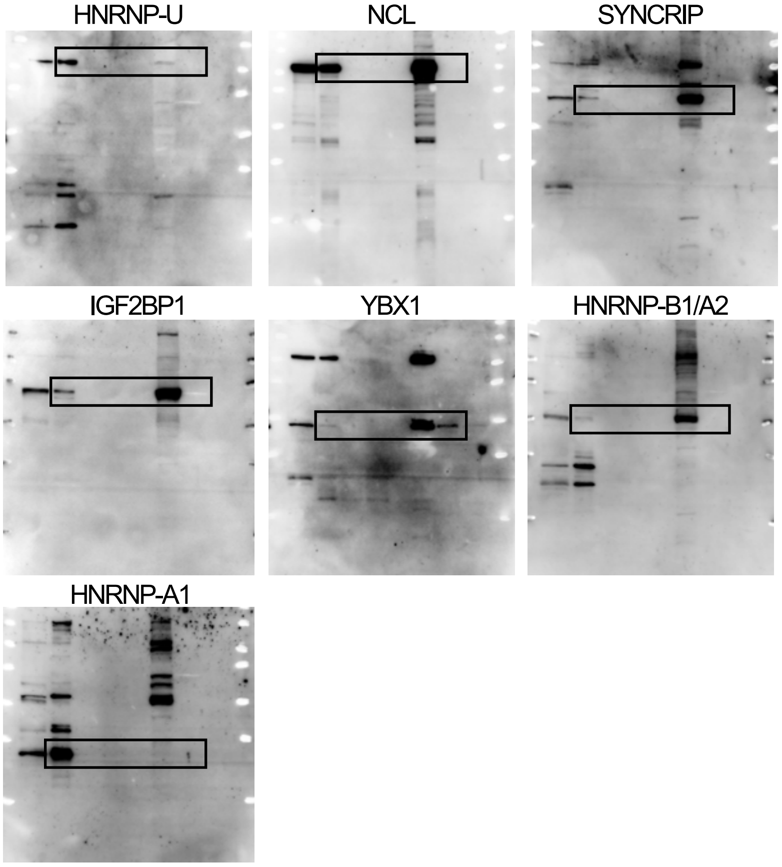

Figure 3c

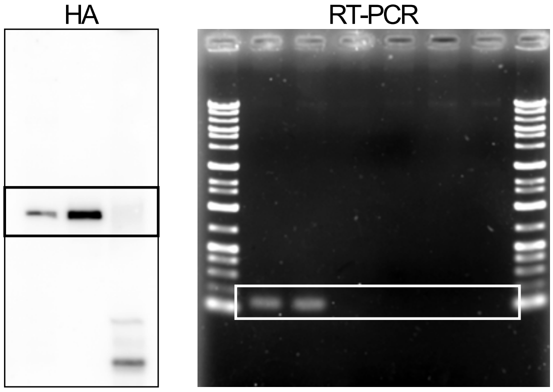

Supplementary Figure 11 | Full-length blot images for the main figures. Full-length northern blotting, western blotting, and gel images for Figure 2b, Figure 3b, and Figure 3c.

Band	Protein name	Locus	Mass	Score	Match	Sequences	emPAI
C1 (115kDa)	Heterogenous nuclear ribonucleoprotein U (Hnrnp U)	AAC26866	87923	265	25	16	0.44
	Protein bicaudal C homolog 1	EDL31961	104971	189	14	12	0.2
	Bicaudal C homolog 1 (Drosophila), isoform CRA_b	EDL31961	104224	163	13	11	0.13
	Unnamed protein product	BAC26122	58230	95	4	3	0.06
	Unnamed protein product	BAB31776	65586	75	5	3	0.05
	Dhx36 protein	AAI38062	113769	74	3	3	0.03
	Protease, serine, 1 precursor	NP_444473	26118	68	2	1	0.13
	Unnamed protein product	BAE40098	39587	42	1	1	0.08
	Cask protein	AAH09740	40806	36	14	1	0.08
	UbcM4-interacting protein 77	AF361001_1	23034	33	1	1	0.15
	Nucleolin	AAH05460	76733	30	2	2	0.04
C2 (105kDa)	Nucleolin	AAH05460	76733	290	30	18	0.8
	Unnamed protein product	BAC26311	76666	290	30	18	0.42
	Heterogenous nuclear ribonucleoprotein U (Hnrnp U)	AAC26866	87837	129	6	6	0.16
	Unnamed protein product	BAB31776	65586	109	8	6	0.4
	Unnamed protein product	BAE35231	87865	107	6	6	0.11
	Protease, serine, 1 precursor	NP_444473	26118	107	3	3	0.18
C3 (70kDa)	Synaptotagmin binding, cytoplasmic RNA interacting protein (Syncrip)	BAA88342	62505	683	69	33	3.9
	Unnamed protein product	BAC37152	54882	598	58	30	3.3
	Protease, serine, 1 precursor	NP_444473	26118	70	1	1	0.13
	Insulin-like growth factor 2 mRNA-binding protein 1 (Igf2bp1)	NP_034081	63411	62	1	1	0.05
	Insulin-like growth factor 2 mRNA-binding protein 2 (Igf2bp2)	NP_898850	65543	53	3	3	0.05
	Eif4g3 protein, partial	AAH47531	128708	46	6	2	0.03
	Anti-diuron immunoglobulin kappa light chain, partial	AAA92993	23853	34	1	1	0.14
	mKIAA0840 protein	BAC98037	57764	33	1	1	0.06
C4 (45kDa)	Y box-binbing protein (YBX1)	CAA40847	35822	145	9	7	0.3
	Unnamed protein product	BAC26122	58230	103	4	2	0.12
	Unnamed protein product	BAB31776	65586	95	3	2	0.1
	Protease, serine, 1 precursor	NP_444473	26118	93	2	1	0.27
	Tcp-10	CAA41161	47082	35	1	1	0.07
	AlphaCP-3	AF176327_1	35936	31	2	1	0.09
	Putative hexokinase HKDC1	NP_663394	102193	30	2	2	0.03
	mCG19088	EDL16344	123759	26	2	2	0.03
N1 (135kDa)	Unnamed protein product	BAC40725	87923	190	14	10	0.25

	Eps8l2 protein	AAH09098	44811	34	1	1	0.07
	Zinc finger, HIT type 3	EDL15753	4715	33	11	1	0.79
	AlphaA-CRYBP1	CAA48762	70173	31	21	1	0.05
N2 (65kDa)	Heterogeneous nuclear ribonucleoprotein L (Hnrnp L)	AAH27206	60085	170	11	8	0.38
	Unnamed protein product	BAB31776	65586	94	2	2	0.05
	Zinc finger, HIT type 3	EDL15753	4715	56	61	2	0.79
	Zinc finger protein 946	NP_932120	61134	36	1	1	0.05
	Cask protein	AAH09740	40806	31	33	1	0.08
	AlphaA-CRYBP1	CAA48762	70173	31	62	1	0.05
N3 (33kDa)	Heterogenous nuclear ribonucleoprotein A2/B1 (Hprnp A2/B1)	AAC26867	35971	169	7	6	0.3
	Heterogeneous nuclear ribonucleoprotein A1 isoform a (Hnrnp A1)	NP_002127	34175	56	3	3	0.1
	Eif4g3	AAH23898	114868	45	7	1	0.03
	BTB/POZ domain-containing protein KCTD1 isoform 1	NP_001136203	96054	32	1	1	0.03
N4 (32kDa)	Heterogeneous nuclear ribonucleoprotein A1 isoform a (Hnrnp A1)	NP_002127	34175	190	8	6	0.74
	Unnamed protein product	BAB31776	65586	116	9	6	0.22
	Y box-binbing protein (YBX1)	CAA40847	35822	110	6	5	0.56

Supplementary Table 1 | MajSAT RNA binding protein candidates searched by MASCOT program. Each band detected specifically in the immunoprecipitates with MajSAT Fw probe was analyzed by LS-MS/MS. For details, refer to the user manual on website (Matrix Science, http://www.matrixscience.com/).
